# Biomimetic Gland Models with Engineered Stratagems

**DOI:** 10.34133/research.0232

**Published:** 2023-09-15

**Authors:** Xiang Lin, Lingyu Sun, Minhui Lu, Yuanjin Zhao

**Affiliations:** ^1^ Department of Rheumatology and Immunology, Nanjing Drum Tower Hospital, School of Biological Science and Medical Engineering, Southeast University, Nanjing 210096, China.; ^2^ Oujiang Laboratory (Zhejiang Lab for Regenerative Medicine, Vision and Brain Health), Wenzhou Institute, University of Chinese Academy of Sciences, Wenzhou, Zhejiang 325001, China.; ^3^ Southeast University Shenzhen Research Institute, Shenzhen 518071, China.

## Abstract

As extensively distributed tissues throughout the human body, glands play a critical role in various physiological processes. Therefore, the construction of biomimetic gland models in vitro has aroused great interest in multiple disciplines. In the biological field, the researchers focus on optimizing the cell sources and culture techniques to reconstruct the specific structures and functions of glands, such as the emergence of organoid technology. From the perspective of biomedical engineering, the generation of biomimetic gland models depends on the combination of engineered scaffolds and microfluidics, to mimic the in vivo environment of glandular tissues. These engineered stratagems endowed gland models with more biomimetic features, as well as a wide range of application prospects. In this review, we first describe the biomimetic strategies for constructing different in vitro gland models, focusing on the role of microfluidics in promoting the structure and function development of biomimetic glands. After summarizing several common in vitro models of endocrine and exocrine glands, the applications of gland models in disease modelling, drug screening, regenerative medicine, and personalized medicine are enumerated. Finally, we conclude the current challenges and our perspective of these biomimetic gland models.

## Introduction

Glands are organs or tissues that secrete or excrete specific substances such as hormones, enzymes, mucus, sweat, and saliva, which are essential for the maintenance of normal functions of various systems and organs in the human body [1–3]. The formation of glands is a complex process involving differentiation and maturation of glandular epithelial cells [4]. Such process begins from the embryonic stage and continues through postnatal growth and maturation, regulated by a complicated network of signaling pathways and transcription factors [5–7]. In general, glands can be categorized into 2 major types: exocrine glands and endocrine glands. Exocrine glands discharge their products into ducts leading to either the body surface or a cavity, such as sweat glands, salivary glands, and mammary glands. Conversely, endocrine glands directly release their products into the bloodstream, including the thyroid gland, pituitary gland, and adrenal gland, followed by transportation to targeted cells throughout the body [8,9]. Because of their importance in maintaining normal physiological processes, the malfunction or disease of glands may induce a broad range of health issues, including diabetes, thyroid disease, and cancer [10–12]. Given that, the construction of biomimetic gland models in vitro is vital for revealing the underlying mechanism of the development and disease progression of glands [13,14]. During the construction process, various demands should be satisfied including the replication of the complex cellular and structural heterogeneity of actual glands, as well as the reproduction of microenvironments containing multiple chemical and mechanical cues [15,16].

Tremendous efforts have been devoted to developing biomimetic gland models from a biological perspective. To build in vitro models that accurately represent the actual glands, it is necessary to select the appropriate cell type referring to glands [17–20]. For example, the mammary gland contains both functional epithelial cells and muscle cells, while the liver consists of liver cells and hepatic stellate cells [21,22]. Based on the gland-derived cell models with secretory function in vitro, pharmacological and toxicological evaluation could be conducted by detecting the corresponding secretory products [23,24]. Although glandular cells can partially replicate certain gland functions in vitro, the simplification of gland models often fails to fully capture the critical shape and function features of the gland tissue [25,26]. In contrast, self-organized organoid models provide a promising solution to this challenge [27,28]. These models are developed from patient-derived stem cells or tumor tissues in a specific 3-dimensional (3D) extracellular environment, allowing the formation of miniaturized organoid models with similar characteristics of real glands, such as microscale size, together with genetic and epigenetic features [19,29,30].

Although bioengineered gland models have achieved great progress in recent years, it is still a challenge to completely reconstruct real dynamic and complex microenvironments of glands. Biomedical engineering provides a new approach to simulate the internal environment of gland tissue, taking advantage of the engineered biomaterial scaffolds and microfluidics [31–34]. In this strategy, the scaffolds with gland similar architectures could provide topological cues to support cell growth and differentiation, while the microfluidic system could simulate the in vivo fluid microenvironment for providing spatial and temporal control of physical and mechanical cues [35–38]. Hence, organ-on-a-chip devices combined with biomimetic scaffolds can accurately reproduce physiological and pathophysiological processes of glands at the organ level [39–43]. Recently, the U.S. Food and Drug Administration has gradually lifted restrictions on animal testing in preclinical drug trials and approved the broader use of human cell-derived organ-on-a-chip systems [44]. Therefore, these accurate biomimetic gland models have been gradually utilized to simulate the generation of different disease models, drug screening, regenerative medicine, etc.

Herein, we reviewed the development of in vitro gland models ranging from engineered stratagems to biomedical applications of them. After describing the methods of culturing mammalian gland-derived cells in different kinds of patterns, we focused on the achievement of functional modeling of gland models based on engineering strategies such as organoid and microfluidic technologies. Next, we introduced several common endocrine and exocrine gland biomimetic models. Subsequently, we outlined how understanding the interplay between structure and function in different glands can be harnessed for biomedical purposes, including tasks like disease modeling, drug screening, and tissue regeneration. Finally, we will discuss the remaining challenges and provide forecast on the future research directions of these biomimetic gland models.

## In Vitro Gland Modeling

Various culture methods have been used to profile and study the glandular microenvironment in vitro [45]. Combined with the transwell culture chamber, the single-layer gland structure can be separated from the culture environment to simulate the niche gland environment [46,47]. Dissociated glandular cells can be incorporated into various stromal scaffolds [48], including collagen gels, matrigel, or decellularized extracellular matrices to generate 3D structures for examining glandular function and structure in vitro [49–53]. Alternatively, cells can be reconstituted without the need of scaffolds to form multicellular aggregates using methods such as hanging droplet systems, U-wells, suspension cultures, etc. [54–56]. These biomimetic gland models generated in vitro include key cell types and exhibit basic functions. Based on the self-organization-based gland models and microfluidic systems, the derived organ-on-chip provides a wider range of microenvironmental complexity.

### Two-dimensional patterns

**Fig. 1.**
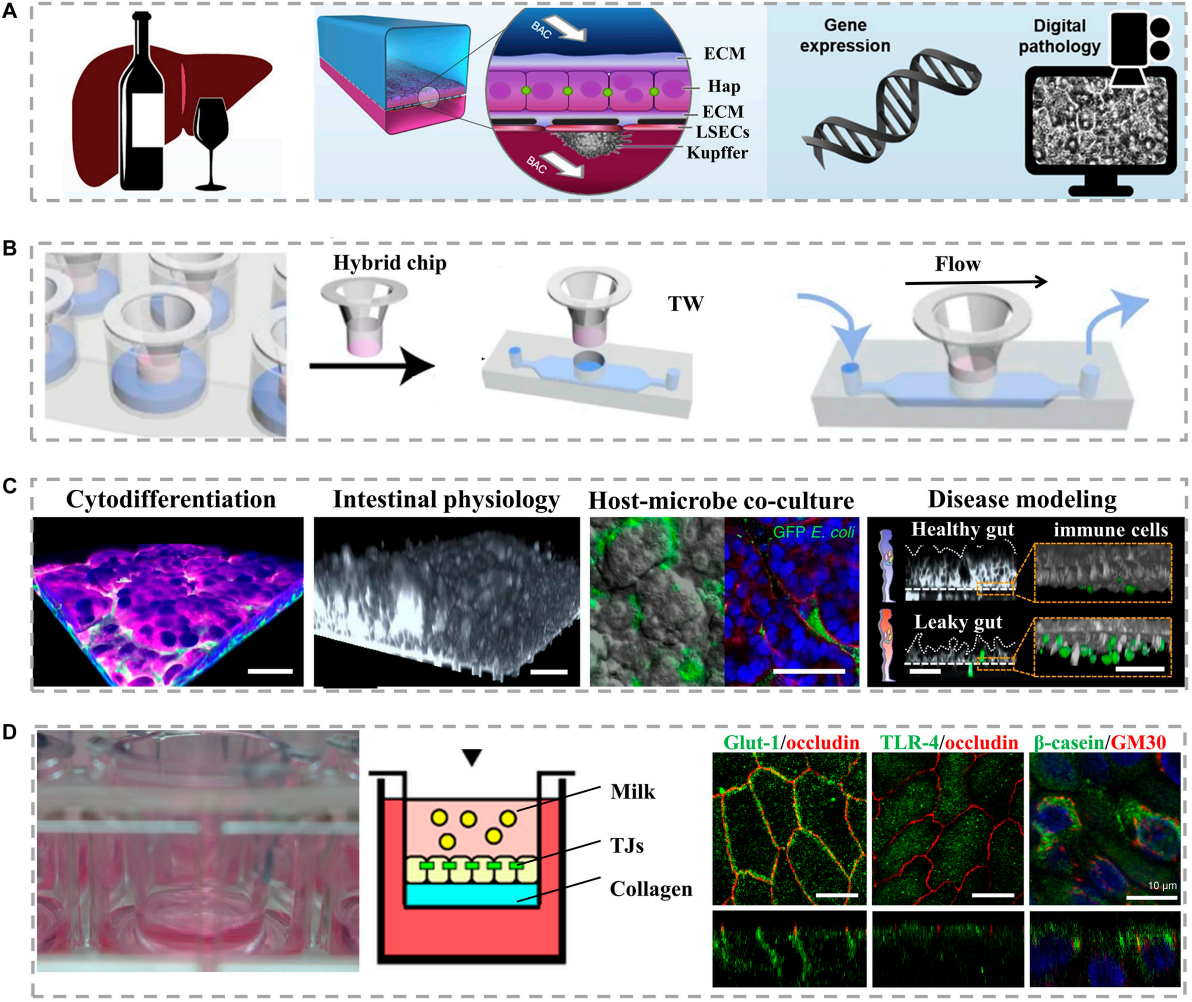
(A) The method of simulating human alcohol-associated liver disease/steatohepatitis by exposing the organ-type liver chip to the blood alcohol concentration related to human [65], Copyright 2021, CellPress. (B) Overall steps of epithelial cell culture on the transwell inserted hybrid chip [66], Copyright 2022, Springer Nature. (C) The application provides an example of the established epithelial layer, which can be used to characterize cell differentiation and physiological research, and establish host microbial ecosystem and disease modeling [66], Copyright 2022, Springer Nature. (D) Schematic images of transwell culture, and the images on the right shows the secretion of mammary epithelial cells in each culture model and the immunostaining [67], Copyright 2020, Wiley-VCH.

As a pivotal role of glands, the examination of secretory function in in vitro glandular models has become a focal point of research [57,58]. Among various culture patterns, traditional 2-dimensional (2D) cell culture has been used not only to study different types of cells in vitro but also to model and test cell absorption, distribution, metabolism condition, and other functions [59,60]. The advantages of the 2D cell model system include convenient operation in the laboratory, lower costs, and wider ethical acceptance compared to using animal models. Considering this, in vitro gland models for hepatotoxicity studies often rely on 2D cell culture [61,62]. Because this method cannot maintain constant concentration of culture activity over a period of time, it is necessary to change the medium regularly to remove accumulated catabolites and renew nutrients for maintaining cell viability and function [63,64]. This allows the preservation of communication between different hepatocytes and liver-specific functions, enabling a wide range of applications, such as acute liver failure, drug interactions, etc. (Fig. 1A) [65]. However, a major drawback of single-layer models is the lack of reliability due to cell dedifferentiation and senescence over time.

Transwell cell culture is an invasion assay technique that enables crosstalk between different layers of cells, by seeding cells in the upper or lower chamber. Because the membrane between chambers is permeable (0.4 to 8 μm), the components in the lower layer of the culture medium could affect the cells in another chamber (Fig. 1B) [66]. Thus, this approach enables improved examination of material transfer, cell–cell interactions between disparate chambers, and related phenomena (Fig. 1C) [66]. This unique culture technique can also be used to study the secretory function of glandular cell monolayers. During lactation, mammary acini produce a diverse array of milk components. The establishment and maintenance of cellular polarity within these acinar structures are critical for the normal lactation pathway of mammary epithelial cells, allowing for the efficient synthesis and secretion of milk constituents. To mimic these processes, the transwell technology can establish a concentration gradient in the same culture system, taking advantage of the tight junction (TJ) between cells to explore the secretion (Fig. 1D) [67]. TJs between monolayer cells prevent the unidirectional diffusion of secreted milk components, together with the external reabsorption of nutrients such as amino acids and lactose by epithelial cells. Apical secretory function with polarity is mimicked in vitro by using TJs to segment the distinct apical and basolateral regions.

### Three-dimensional scaffolds

**Fig. 2.**
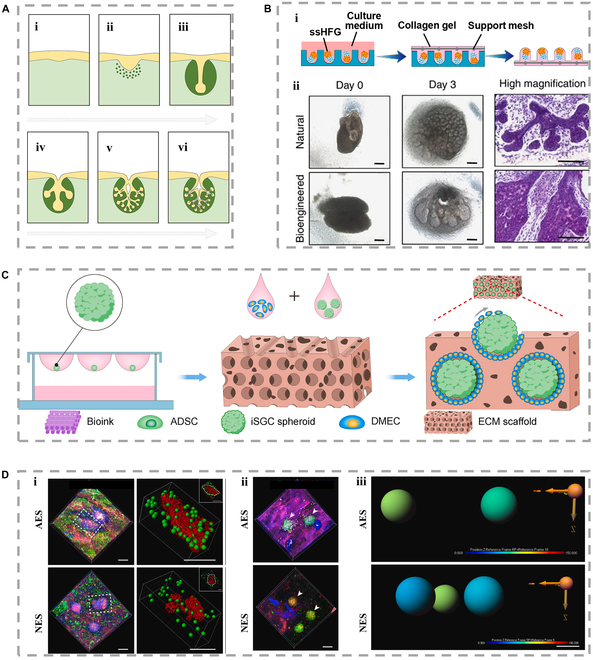
(A) Basic steps of morphogenesis in salivary gland [68], including (i) pre-bud, (ii) early bud, (iii) initial bud, (iv) initial gland, (v) immature gland, and (vi) terminal gland, Copyright 2021, Elsevier. (B) Diagram (i) shows that hair follicle germs are embedded in collagen gel for transplantation on the 0 and 3 days of organoid culture [69], Copyright 2018, Elsevier. (ii) Enzyme contrast images of salivary gland (top) and bioengineered gland (bottom); on the third day of organ culture, salivary glands were analyzed with hematoxylin and eosin staining [190], Copyright 2013, Springer Nature. (C) Schematic diagram of establishing biomimetic sweat glands–vaculatory interaction model in vitro. (D) 3D reconstruction of (i) SG–vaculatory interaction model based on confocal z-stack. Dermal microvascular endothelial cells (DMECs) are replaced by the green sphere, and the induced sweat gland cell (iSGC) sphere is replaced by the red rough surface. (ii) The depicted fluorescent image and three-dimensional reconstruction showcase the iSGC globule in a representative manner. (iii) The arrow represents the 3D image analysis of each iSGC sphere [80], Copyright 2021, KeAi.

Modeling the intricate structure of glands outside of their natural environment poses a challenge due to their highly branched and multicellular organization (Fig. 2A) [68,69]. To replicate this complexity, the extracellular matrix (ECM) provides a 3D structure comprising decellularized proteins, both fibrous and non-fibrous, that surround cells in multicellular organisms (excluding circulating fluid) [70,71]. The ECM plays a critical role in both embryonic development and cellular homeostasis, and has wide-ranging implications for tissue engineering and regenerative medicine [72]. Unlike 2D layers, the use of 3D scaffolds enables the recreation of the gland's native ECM properties, including their physical, biochemical, and mechanical features, which further affects cell growth and function [73,74]. The materials used to prepare 3D scaffolds are usually collagen, alginate, and other hydrogels [75–77]. These hydrogels are polymers that retain large amounts of water within their 3D structure, which is crucial for maintaining the integrity of cells and tissues. To achieve in vitro mimicry of the ECM, various hydrogels, including matrigel and collagen derivatives, have been employed to cultivate mammalian cells. Hair follicle morphogenesis is a typical model of glandular development. Kageyama et al. [69] reported that they achieved large-scale in vitro fabrication of cells through self-assembly in collagen. The randomly distributed unicellular aggregates were formed during the first 3 days of the culture period, which then separated spatially from each other, exhibiting morphological features typical of hair follicles (Fig. 2B).

In particular, the emerging 3D bioprinting technology could combine stem cells with differentiation potential to generate massively replicable glandular spheroids [78,79]. For instance, dermal microvascular endothelial cells were employed to create vascular niches by crafting a sacrificial template from poly (ε-caprolactone) (Fig. 2C and D) [80]. The model enables the in vitro development of vascularized gland morphogenesis that closely mimics physiological conditions. With the combination of microarrays, such biomimetic model could further be employed to study the development process of glands in vitro and the mechanism of reciprocal regulation. However, the pre-structured scaffold's architecture may impede the mimicry of tissue and glandular morphogenesis. In addition, the generation of dynamic luminal structures within these scaffolds remains challenging. These problems pose a crucial hindrance to glandular cell morphogenesis and physiological secretory functions, which necessitate the presence of fluid shear forces.

### Microfluidic platforms

**Fig. 3.**
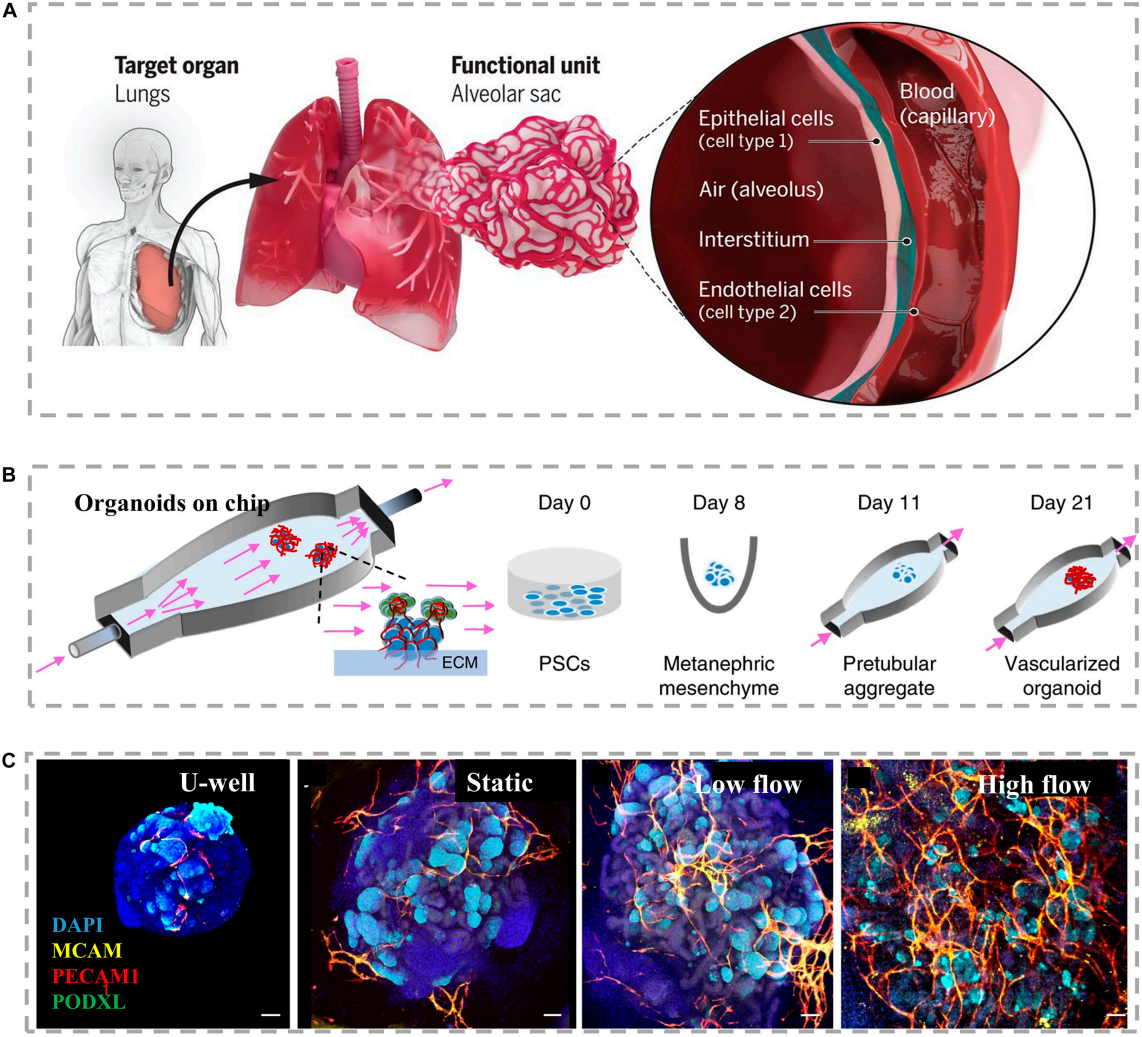
(A) The reduction analysis of the target organ (lung) and the functional unit of alveolus composed of epithelial cells and endothelial cells, separated by stroma [85], Copyright 2019, Science. (B) The developing renal organs are placed on an engineering microfluidic platform and a perfusion microfluidic chip [93], Copyright 2019, Springer Nature. (C) Confocal images showcasing vascular markers in fully scaffolded organs cultured under varying conditions such as static U-shaped hole, static engineered extracellular matrix (ECM), low flow stress, and high flow stress [93], Copyright 2019, Springer Nature.

Microfluidic platforms have emerged as powerful tools for creating in vitro models of various biological systems, including gland models. These platforms enable researchers to mimic the complex microenvironment of glands and study their functionality, response to stimuli, and disease mechanisms in a controlled and precise manner. The proper functioning of glands is inseparable from a strong blood circulation system [81,82]. In order to construct glands with accurate physiology, it is important to examine the multilevel structure of the target gland, as glandular cells do not function well in an unchanging culture substrate [83,84]. For example, alveoli consisting of distinct epithelial and endothelial cells include different microenvironments, such as the gas–liquid interface and liquid–liquid cross-section (Fig. 3A) [85]. For the construction of circulatory systems, microfluidic culture technology offers the possibility to address all these challenges, whether in 2D or 3D culture. Organs-on-a-chip devices allowed the culture of living cells under fluid flow, which can recapitulate physiology and pathophysiology at the organ level with high fidelity. These physiological processes can be explored in different glandular culture models with the aid of microfluidic systems.

The incorporation of microfluidics into 3D cell culture systems enables the delivery of controlled mechanical, chemical, and biological signals [86–88]. Several methods have been suggested for controlling the spatial and temporal characteristics of cellular microenvironments [89]. These techniques encompass adjustments in stiffness, micropatterning, 3D arrangements, and concentration gradients [90–92]. Specifically, cultured endothelial cells can be induced to differentiate in a chamber, through applying physiological shear stress and specific signaling stimuli. Similarly, functional glandular cells seeded on viscoelastic ECM gels can self-organize into polarized acinar structures [60]. Fluid shear stress brought about by fluids can promote the maturation of glandular tissues in vitro, while the development and function of glands are restricted in static culture (Fig. 3B) [93]. For example, the gene expression with the assistant of microfluidics observed in organoids composed of renal filtration-associated epithelial cells reflects the same condition during filtration in vivo (Fig. 3C) [93]. The maturity of the adult kidney is relatively low. Under high fluid flow, the cultivation of in vitro kidney organoids results in improved vascularization during the process of nephrogenesis.

### Self-organizing organoids

The progress in stem cell therapy and tissue regeneration has facilitated the development of self-organizing organoids that recapitulate the structure and function of native tissues [94,95]. These 3D structures arisen from the spontaneous assembly of stem cells under optimized culture conditions could promote cell–cell and cell–ECM interactions [96–98]. The growth factors, ECM components, and mechanistic cues that drive organoid formation are carefully selected and tailored to the specific tissue type being modeled [99]. Besides, self-organizing organoids provide several advantages over traditional 2D cell culture and animal models for investigating glandular diseases and drug responses [100,101]. Firstly, they better replicate the intricate 3D structures and cellular heterogeneity of native tissues, enabling more precise modeling of disease mechanisms and drug effects [102,103]. Secondly, patient-derived stem cells can be utilized for developing personalized medicine approaches, and the investigation of rare or genetic disorders that are difficult to simulate in animals [104,105]. Lastly, self-organizing organoids can be readily scaled up for high-throughput drug screening and toxicology testing [106,107].

**Fig. 4.**
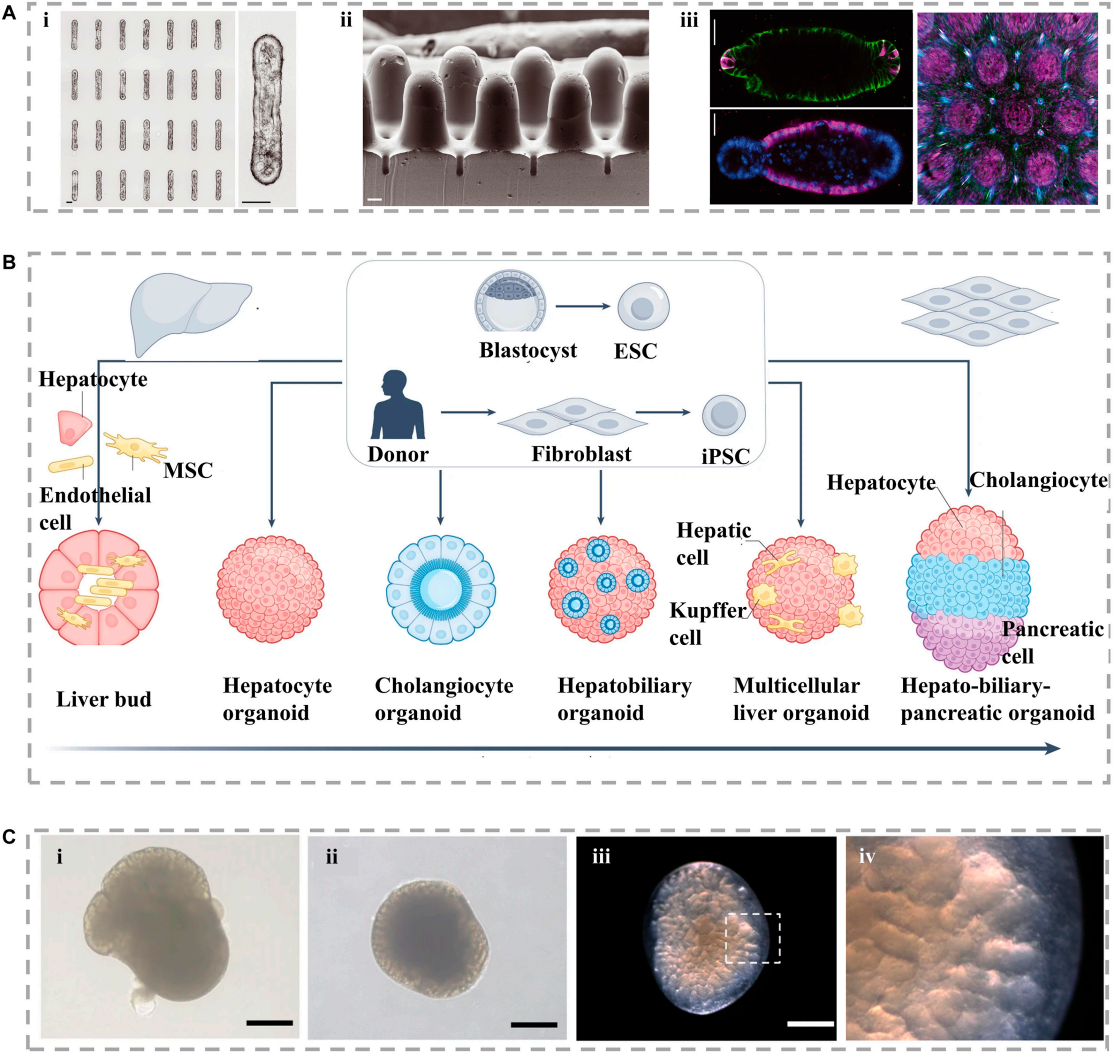
(A) Through the external control of engineering microenvironment on organoid development, based on the micro-engineering method, achieve precise micrometer-scale control over the size and shape of (i) organoids, (ii) predictably and consistently control the geometric configuration of organoids to create patterns, and (iii) employ geometry-mediated organoid models to generate a macro-intestinal surface featuring a crypt-villous structure [109], Copyright 2022, Science. (B) Liver organs are derived from liver progenitor cells, primary hepatocytes, fibroblasts, or human pluripotent stem cells, encompassing a spectrum of cell sources [108], Copyright 2022, Springer Nature. (C) (i) One gland phase contrast image on the 60th day. (ii) Representative images of human induced salivary glands separated on the 80th day. (iii) Representative stereo microscope image of human induced salivary glands on the 80th day. (iv) Enlarged image [110], Copyright 2022, Springer Nature.

Self-organizing organoids have been used to mimic a range of glandular tissues, including liver, pancreas, salivary glands, and mammary glands. These organoids can recapitulate key aspects of gland development and function, including tissue architecture, cellular heterogeneity, and physiological responses to hormones and drugs (Fig. 4A and B) [108,109]. They are spherical collections of self-organizing cells that resemble natural tissues and exhibit organ function and 3D structure. Organoids have several inherent advantages over traditional models, including the establishment of macroscopic structures similar as in vivo tissue in complex 3D environments. Besides, the crosstalk between organoid cells can enhance the similarity to podocytes in vivo compared with traditional 2D culture methods. The 3D environment is critical not only during the differentiation process but also in maintaining the differentiated state, as organoid-derived podocytes de-differentiate in a 2D environment. Interestingly, when studying the functional effects of some viruses on the gland, human organ model systems have some unique advantages compared to animal models. Since the COVID-19 pandemic, human airway organoids faithfully mimic human airway epithelial cells, thus providing a platform for rapid detection of infectivity of emerging influenza viruses (Fig. 4C) [110]. A laboratory-generated salivary gland model derived from human-induced human induced pluripotent stem cells (hiPSCs) was found to be permissive to COVID infection. The findings indicate that salivary glands exhibit developmental characteristics similar to those observed during embryonic stages and can serve as a valuable tool for investigating gene function in development. Notably, the in vitro salivary gland model sustained replication of the COVID virus, suggesting its potential utility in studying viral infection mechanisms. Therefore, organoids are promising models for studying glands in vitro.

## Endocrine and Exocrine Gland Models

Glands are categorized into endocrine or exocrine types: endocrine glands release their secretions, including hormones, directly into the bloodstream, whereas exocrine glands secrete substances into body cavities (such as the mouth) or external surfaces (like the skin) [111–113]. In recent years, in vitro glandular models have garnered extensive attention as a substitute for animal models in investigating glandular diseases [114,115]. These models are highly sought-after for their potential in drug development and for studying glandular physiology, as they provide a controllable and reproducible environment for investigating the physiology and pathology of glands in a setting that mimics the human body [116,117]. The process of constructing engineered gland models involves a combination of techniques including cell culture, biomaterials, and microfabrication. Various methods have been employed to create glandular structures such as 3D printing, microfluidics, and self-assembly, which offer the ability to investigate glandular secretion, morphogenesis, and cellular interactions [19,118]. Furthermore, the incorporation of human stem cells and patient-derived cells in these models has demonstrated promising outcomes for simulating glandular diseases [119,120]. However, further research is necessary to enhance the fidelity of these models to in vivo systems. Here, we provide an overview of current commonly established in vitro glandular models for both endocrine and exocrine glands (Table).

**Table.** The commonly established *in vitro* glandular models.

Glandular type	Glandular models	Engineering strategies	Applications
Endocrine gland	Thyroid	Organoids	Hypothyroidism [117]
Endocrine gland	Medullary thyroid	Two-dimensional patterns; three-dimensional scaffolds	Modelling of medullary thyroid carcinoma; drug screening [118]
Endocrine gland	Adrenal	Organoids	Physiologic adrenal remodeling; pathologic alterations [125]
Endocrine gland	Uterus	Two-dimensional patterns; microfluidics	Angiogenesis and hormone response; simulate the proliferation and secretion of menstrual cycle [126]
Endocrine gland	Gonad	Organoids	Human gonad development [127]
Endocrine gland	Thymus	Organoids; three -dimensional scaffolds	Reconstitution of a functional human thymus [129]
Exocrine gland	Liver	Organoids; microfluidics	Hepatic fibrosis models [135]
Exocrine gland	Salivary	Organoids	Model SARS-CoV-2 infection and replication [108]
Exocrine gland	Sebaceous	Three-dimensional scaffolds; organoids	Skin biology; model disease [138]
Exocrine gland	Sweat gland	Three-dimensional scaffolds; organoids	Recapitulated damaged skin [139]
Exocrine gland	Mammary gland	Organoids	Breast cancer [140]
		Three-dimensional scaffolds	Branching and alveologenesis of mammary gland [141,142]

### Endocrine gland

#### Thyroid

**Fig. 5.**
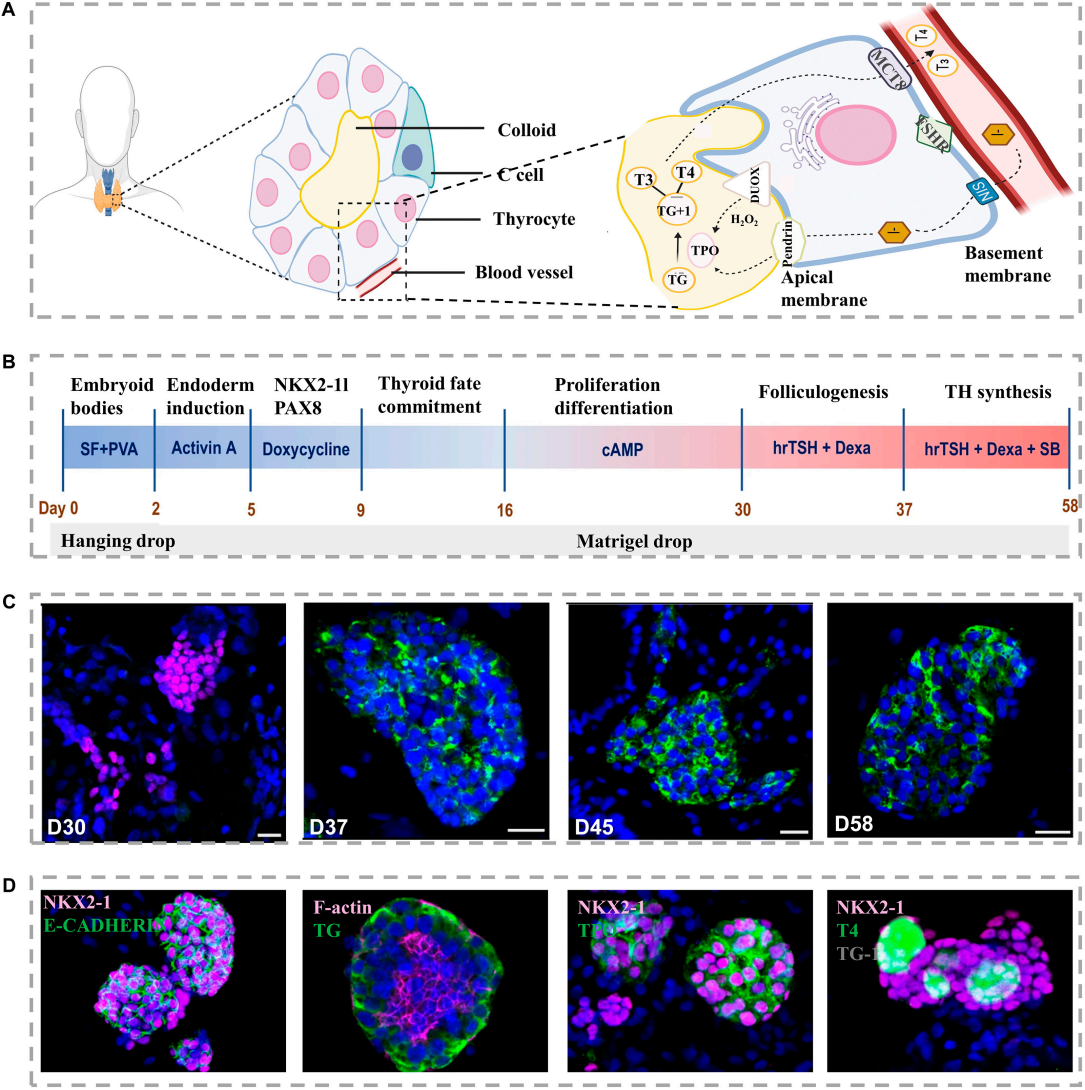
(A) The basic structural units of the thyroid gland and its hormone synthesis process [121], Copyright 2022, Terms. (B) Strategy for thyroid follicle differentiation [122], Copyright 2022, Springer Nature. (C) Immunostaining revealing thyroid differentiation and cellular tissue changes [122], Copyright 2022, Springer Nature. (D) On the 58th day of the differentiation protocol, visual insights were captured through confocal immunofluorescence images [122], Copyright 2022, Springer Nature.

The thyroid glands are mainly consisted of follicular cells and parafollicular cells (C cells) [121]. Upon receiving the stimulation of thyroid-stimulating hormone (TSH) secreted by the thyroid gland, the follicular cells in the inner layer would synthesize iodized thyroglobulin (Fig. 5A) [121]. The generated TSH would be degraded in the thyroid cells, and the remaining hormone enters the blood circulation. Former research on the thyroid mostly focused on hormone metabolism and iodide metabolism in animal models. In recent years, in order to simulate this metabolic process in an in vitro thyroid model, it is crucial to establish cultures derived from monolayer cells, or supplemented with TSH-containing medium and generate 3D acinar structures. Romitti et al. [122] have reported a novel technique, as depicted in Fig. 5B to D; they utilized stem cells with reprogrammed and modified signaling pathways to produce a functional human thyroid gland in vitro that can mimic the gland's function. The researchers demonstrated the creation of transplantable thyroid organoids derived from human embryonic stem cells. These organoids successfully reinstated plasma thyroid hormones in mice with an absent thyroid gland. Notably, follicular cells can form 3D acinus-like structures regardless of whether the 3D culture substrate is matrigel or collagen; although organoids have been successful in replicating the complex structures and functions of various organs, they still lack a crucial component—vascularity. To ensure that thyroid organoids are truly functional, both further vascular development and indeed enhanced maturation of the organoids are required. To promote vessel formation after (xeno) transplantation, vessel-like structures could be constructed, and in vitro biomaterials can also be utilized to create 3D culture systems that incorporate both tissue-derived organoids and vascular networks.

Tumor gland models can help researchers better understand cancer biology, test potential therapies, and develop new treatment strategies. Although there are fewer cancers generated by C cells, their prognosis is worse than that of small papillary carcinomas due to their low degree of differentiation. Medullary thyroid cancer makes up around 5% of all thyroid cancer cases, predominantly impacting C cells rather than follicular cells. So far, studies on the establishment of thyroid C cells in vitro are limited. In this scenario, Kwaku detailed an approach to construct a model for thyroid C cells. Here, human embryonic stem cells undergo progressive differentiation into cells resembling thyroid C cells, both in monolayer and within 3D matrigel culture environments [123]. The procedure entails a stepwise application of interferon as part of hormone treatment, mirroring the developmental sequence of the embryonic thyroid. Moreover, aside from quantifying the expression of C cell and related follicular lineage markers using qPCR (quantitative polymerase chain reaction) and immunolabeling, these cells exhibited functional attributes. This was evidenced by their ability to release calcitonin upon calcium stimulation, a trait confirmed through enzyme-linked immunosorbent assay during in vitro experiments.

#### Adrenal gland

In recent years, although researchers had gained much progress about human adrenal development and function from human (genetic) disease, the studies in adrenal gland have been hampered due to its distinctive structure and functions [124–126]. Only higher primates like monkeys share a similar structure and function of the adrenal cortex to humans, whereas rodents exhibit marked structural and functional differences. Therefore, there are many limitations when using animals such as mice as models for building in vitro models. Much of the current knowledge about adrenal development comes from mouse models, yet we still do not understand the intricate details of fetal adrenal development and function. According to existing studies, the adrenal gland is a polyendocrine organ with a mesenchymal cortex producing steroids and an endocrine medulla producing catecholamines of neuroendocrine origin [127–129]. Supported by a pool of stem/progenitor cells, this gland has a strong regenerative capacity, enabling it to maintain physiological regulation and meet hormonal demands.

Until now, benefiting from the single-cell sequencing technologies, spatiotemporal gene expression profiling of human fetal material has provided additional insights into developmental pathways. Besides, it is also a hot topic to construct in vitro models by combining various biomaterials and reprogrammed hiPSCs. Poli et al. [130] have created an adrenal gland model using fetal adrenal specimens from various gestational cycles. These cells demonstrated the ability to maintain their developmental processes in vitro and expressed markers indicative of primitive fetal organs, including the zona pellucida. Additionally, the cells spontaneously organized into 3D organ-oids with structural similarities to fetal glands. While this in vitro human adrenal system is preliminary in nature, it represented a unique opportunity for investigating the pathophysiological mechanisms underlying rational adrenal remodeling, organ hypoplasia and hyperplasia, and cancer.

#### Gonad

**Fig. 6.**
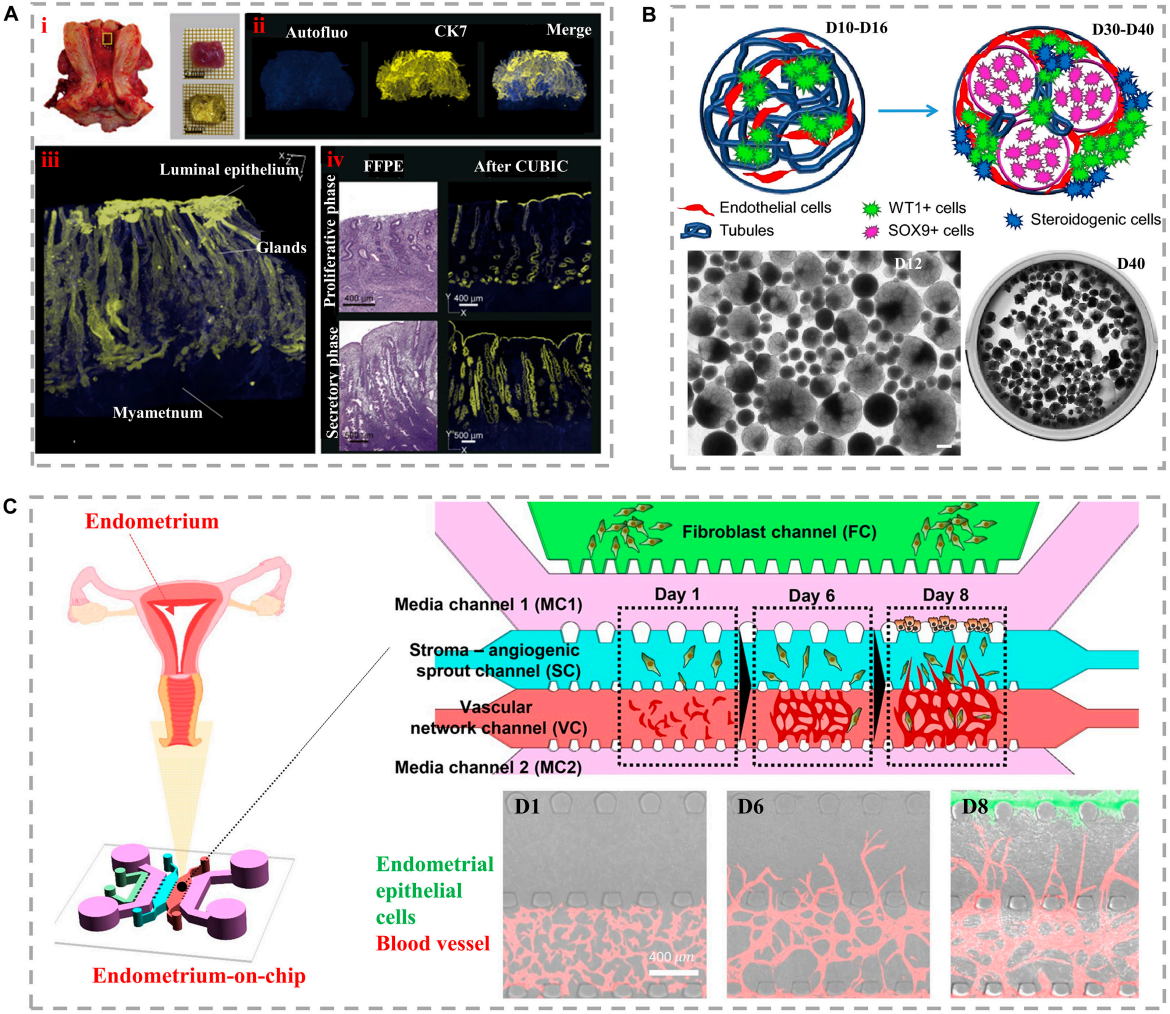
(A) E2-targeted patient. (i) Site of human anatomy, and clearing performance of E2-targeted human anatomy, and specific clearing performance. (ii) 3D images of tissue from subject E2. (iii) Histologically enlarged 3D image reveals a large amount of glandular and luminal epithelial skin layer. (iv) Microscopic H&E-stained image and double-layered image comparison after tertiary cleaning [120], Copyright 2021, Elsevier. (B) Indicative and representative images of the growth process of medial organs in a long-term period [131], Copyright 2022, Springer Nature. (C) Overview of membrane microenvironmental indicative images [132], Copyright 2022, Oxford University Press.

Prior knowledge about human gonads was largely obtained through histological analysis of tissue sections. Recently, Yamaguchi et al. [120] (Fig. 6A) used modeled slices of human endometrial tissue to study its morphology using 3D immunohistochemistry and layer analysis. Their findings showed that endometrial glands formed a tufted network in the basal layer and extended horizontally along the muscle layer. This in vitro approach can be used to assess uterine diseases such as adenomyosis and observe the affected tissue's 3D morphology. However, conducting research on human embryonic gonads is hindered by ethical and legal constraints and limited tissue availability. To overcome these limitations, in vitro models using stem-cell-derived gonads are needed to further explore the mechanisms underlying the development of gonads, the appearance of distinct cell types, and the shaping of tissues.

Human pluripotent stem cell (hPSC)-based organoids are 3D structures that mimic the complexity and functionality of human organs and tissues. These structures have shown great potential for constructing developing organs and tissues, as well as for modeling human diseases. In recent years, hPSC organoids have emerged as a powerful tool for studying the early stages of human reproductive development. With specific culture conditions, hPSCs can differentiate into different cell types, including those that form the gonads. This offers a unique opportunity to study the pathways that lead to the formation of male and female reproductive organs. Pryzhkova et al. [131] (Fig. 6B) presented a novel approach to studying tissue and organ development in vitro using 3D organoid technology of hPSCs. The researchers used suspension bioreactors to culture hPSC organoids, which allowed them to simulate the early stages of human reproductive development. The organoids were able to self-organize and differentiate into cells that express characteristic gonadal markers, indicating the potential of organoid technology for simulating gonad development. In addition, the researchers studied the influence of vascular cells on testis-specific tissues and successfully detected Hedgehog-BMP regulatory signals. This is important because these signals play a critical role in gonad development. The results showed that activated cells expressing tissue-specific morphogenesis also emerged in culture, indicating that the organoid technology is capable of simulating complex organogenesis. The ability to study the development of human reproductive organs using hPSC organoids has important implications for understanding the underlying mechanisms of reproductive disorders and for developing new therapies to treat them. In the future, this technology could also be used to generate transplantable tissues and organs for patients who require them.

The precise role of cervical epithelium in maintaining cervical integrity during infection and inflammation in gynecological diseases remains poorly understood. To investigate cell–cell interactions, Ahn et al. [132] (Fig. 6C) developed an endocervical epithelial organ-on-a-chip (EOC). The system consisted of 2 co-culture chambers connected by microchannels, allowing for the recapitulation of both endocervical and epithelial structures. Furthermore, the team evaluated the interactions between cells from different regions, together with their influence on maintaining cervical integrity in response to inflammatory stimuli. Co-culturing of extracervical and endocervical cells facilitated the migration of both types of epithelial cells within the microchannels. In comparison with the untreated control group, the inflammatory factor stimulation group demonstrated increased apoptosis, necrosis, and senescence of cervical epithelial cells, as well as increased production of pro-inflammatory cytokines. The EOC system serves as an in vitro model that mimics the exocervical and cervical epithelial regions of the cervix, which has the potential to be a powerful tool for research in obstetrics and gynecology. For example, it can be used to study cervical remodeling during pregnancy and childbirth, as well as the dynamics of cervical epithelial cells in benign and malignant lesions of the cervix.

#### Thymus

**Fig. 7.**
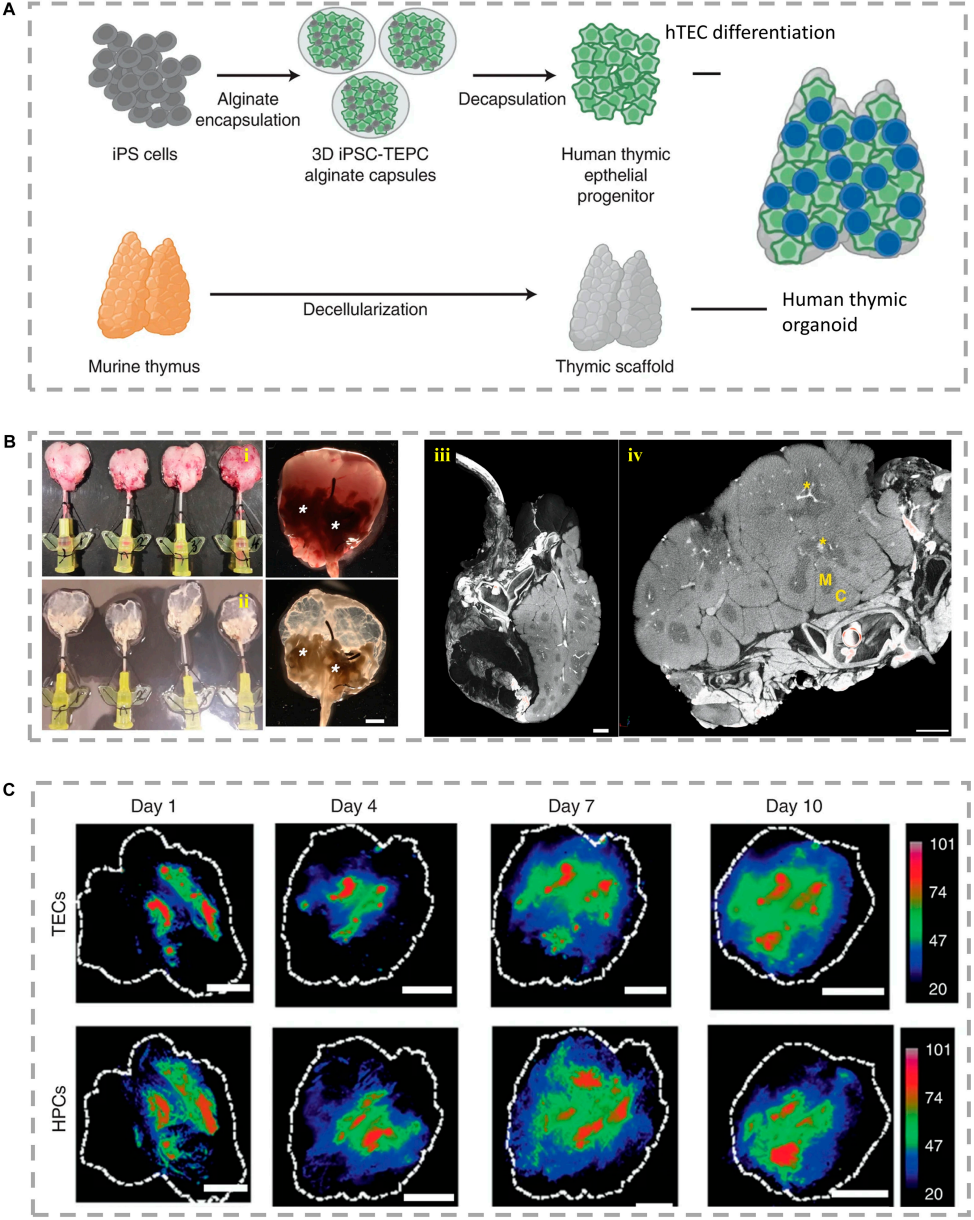
(A) Schematic diagram of human iPSC-thymus organ construction [133], Copyright 2022, Springer Nature. (B) (i) The general appearance of the rat thymus before and (ii) after acellular of the rat thymus. Insert a 24 G sleeve into the carotid artery for perfusion of detergent and enzyme solution. The asterisk indicates extrathymic tissue, allowing thymic tissue and cannula to be connected through large blood vessels. (iii) The microscopic CT image of the rat thymus after intubation shows (iv) extrathymic tissue, large blood vessels, and 24 G intubation enter the artery [134], Copyright 2020, Springer Nature. (C) Imaging of the whole scaffold showed that the distribution of Did-labeled CD34^+^ TECs and DiR-labeled iPSC-TECs changed within 10 days of culture [133], Copyright 2022, Springer Nature.

The thymus is a major lymphoid organ located in the upper mediastinum above the heart, which generates a diverse population of T cells to protect the body from pathogens (Fig. 7A) [133]. Campinoti et al. [134] used an engineered approach to generate in vitro thymic scaffolds in which the hematopoietic and functionalized fractions were hPSCs (Fig. 7B). The researchers created a scaffold composed of 2 cell types that can be expanded in a lab setting for extended periods. By combining thymic stromal cells and decellularized thymic ECM, they were able to reconstruct the thymus' natural anatomical surface. This structural modification enhanced T cell function and promoted the development of mature T cells in immunodeficient mice after transplantation. To summarize, constructing these in vitro models has practical implications for treating both innate and acquired immune diseases.

The development of thymus-based drugs for immune diseases is an emerging field. While xenogeneic animals are commonly used in preclinical research and drug discovery to mimic the human immune system, crafting operational human T cell compartments in experimental settings presents challenges due to variations between human and mouse thymus tissue. While transplanting human fetal thymus tissue into mice has shown potential for fostering robust T cell development, constraints related to tissue availability and ethical considerations hinder its widespread application. A recent investigation led by Zeleniak et al. introduced an alternative strategy involving human thymic organoids generated from induced pluripotent stem cells (iPSC-thymus). These organoids facilitated the efficient generation of substantial functional human T cell populations, capable of dampening humoral immune reactions, including inflammatory responses stemming from T cell receptor interactions (Fig. 7C) [133]. The authors outlined the feasibility of T-cell-mediated personalized immunotherapy based on individual patients. With further protocol refinement, it is possible that artificial thymuses could be bioengineered from patients' own iPSCs to treat immunodeficiency diseases in the future.

### Exocrine gland

#### Liver

**Fig. 8.**
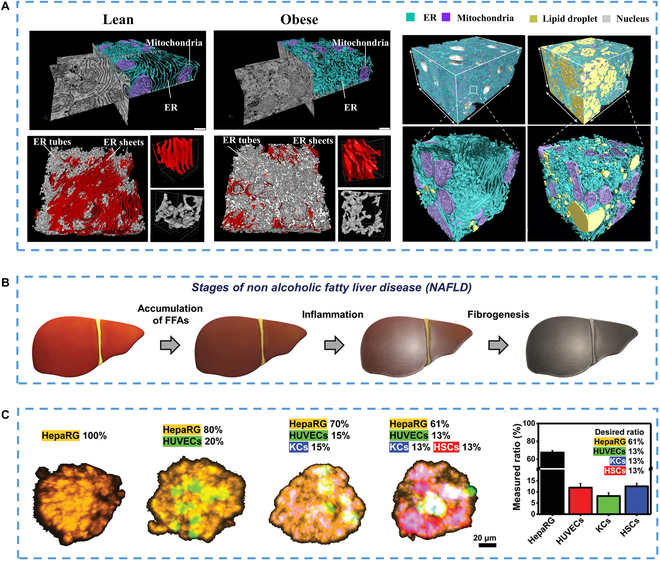
(A) Partially reconstructed segmented endoplasmic reticulum and mitochondria from the original focused ion beam scanning electron microscopy (FIB-SEM) images of liver cells from lean and obese mice. On the right is the automatic segmentation of liver volume from lean and obese mice based on convolution neural network [138], Copyright 2022, Springer Nature. (B) Schematic diagram of liver fibrosis progression driven by non-alcoholic fatty liver disease and bioengineered multiple liver microtissues [139], Copyright 2021, Wiley-VCH. (C) On the 4th day, the representative confocal images of bioengineered multicellular liver microtissues (BE-MLMs) among the groups and the corresponding measurement scores of each cell type contained in BE-MLMs composed of 4 different types of cells [139], Copyright 2021, Wiley-VCH.

Metabolic diseases are becoming a major threat to health in modern society. In particular, the emerging obesity problem also reflects the increase of metabolic diseases, which is usually associated with abnormal liver metabolism [135,136]. Although the metabolism of different types of cells at the tissue level has been reported in succession, the specific structure and spatial morphology of many cells and organelles may also be related to metabolism at the single-cell or even subcellular level [137]. The latest research shows that the subcellular structure of the liver is quite different under the conditions of health and obesity, where structural regulation may be a prerequisite for metabolic programming [108]. For example, studies have shown the effect of increased endoplasmic reticulum tablets on the metabolism of obese mice (Fig. 8A) [138]. The metabolic factor that produces obesity is insulin resistance, which may cause serious lipid buildup in the liver, disrupting normal liver metabolism. Researchers have developed liver organ models using a variety of stem cell types, including adult stem cells, primary stem cells, and hPSCs. These platforms provide new opportunities to analyze the potential mechanism of metabolic diseases and develop personalized drugs.

In addition to obesity, high levels of alcohol consumption would lead to the prevalence of alcoholic liver disease that involved changes in liver function. To prevent this, inhibiting the process from fatty liver to fibrosis to cancer is also a thorny problem. Cho et al. [139] developed a liver-on-a-chip platform using bioengineered multicellular liver microtissues (Fig. 8B and C), incorporating 4 major types of hepatocytes (hepatocytes, endothelial cells, Kupffer cells, and stellate cells) to model human liver fibrosis resulting from nonalcoholic fatty liver disease (NAFLD). By including stellate cells in a fat-supplemented liver-on-a-chip model, they were able to observe elevated inflammatory responses and upregulation of fibrotic markers. This model provides a more natural representation of the pathology and chronology of disease compared to the transforming growth factor-induced liver fibrosis model, which can help in better understanding the mechanism of liver disease fibrosis progression and identifying new drug targets.

#### Salivary

**Fig. 9.**
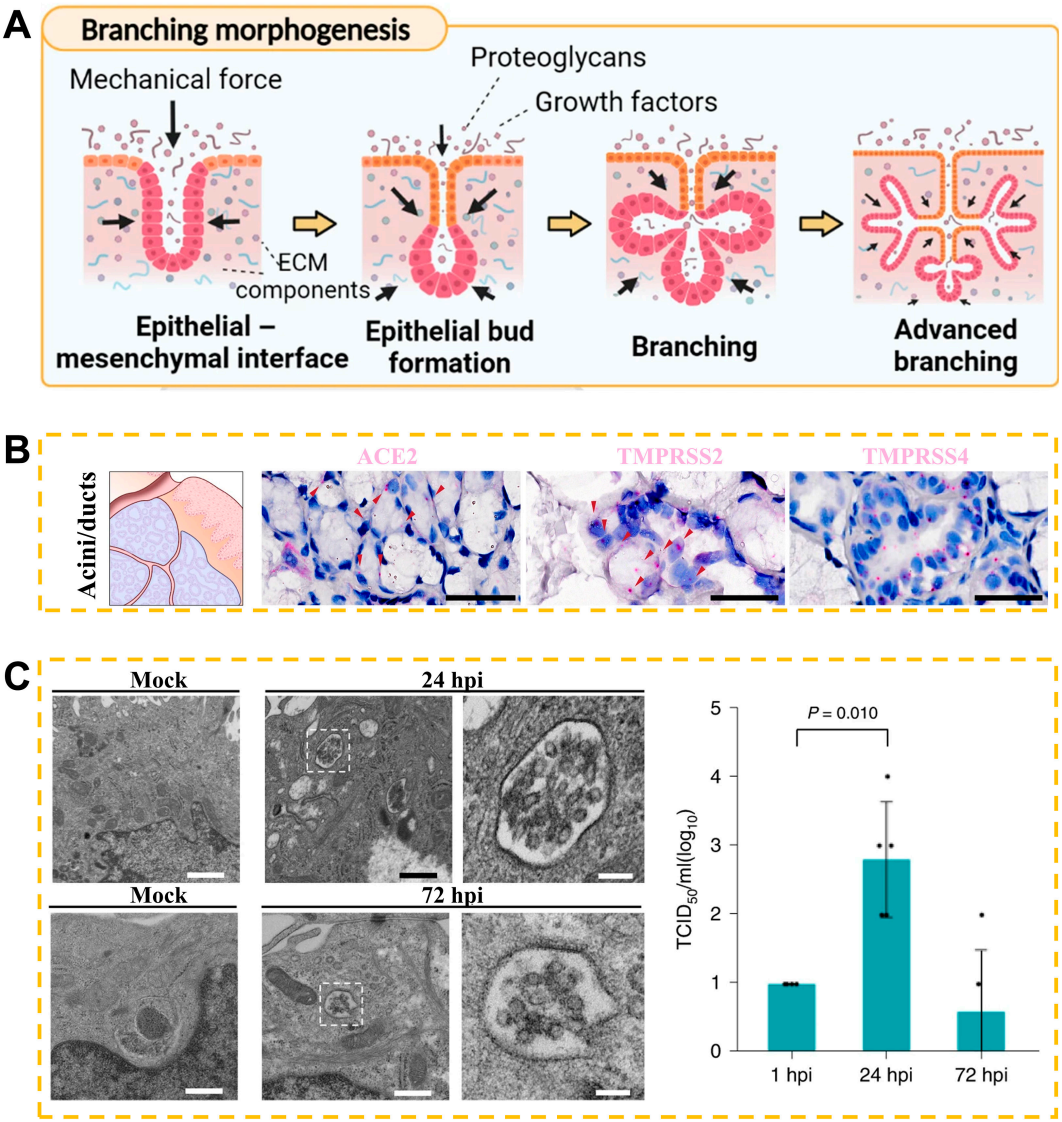
(A) Schematic diagram of branching morphogenesis of salivary gland [140], Copyright 2022, Springer Nature. (B) The mRNA expression in gingiva confirmed by using gingival tissue sections of healthy volunteers [16], Copyright 2022, Springer Nature. (C) Transmission electron microscope images of uninfected (simulated) and infected HISGs. The vesicles in the infected cells are filled with virus particles. On the right is the 50% tissue culture infectious dose (TCID_50_) detection of SARS-CoV-2 [141], Copyright 2022, Springer Nature.

As the branching process occurs, the ECM composition undergoes regional variation and plays a direct role in the maturation of salivary glands (Fig. 9A) [140]. Saliva has many essential functions, including helping to secrete digestive juices and maintaining human health through the secreted water, mucus, electrolytes, antimicrobial compounds, and various enzymes. As the first line of defense against many pathogens that enter through oral defenses, saliva can indicate a person's clinical condition. Researchers have employed various tissue engineering approaches to restore salivary secretion (Fig. 9B) [16], such as self-organization, low-adhesion culture plates, and 3D culture systems, to generate functional salivary gland organoids. Designing a fully functional salivary gland requires complex interactions between various cell types, including acini, ducts, and myoepithelial cells, as well as providing appropriate hormonal stimulation and vasculature to the gland. Therefore, engineered salivary glands must contain unique components and functions. For this purpose, understanding the mechanisms leading to the loss of secretion and involvement in the regeneration of functional organs remains the focus of current and future research.

It is anticipated that organoid culture models will prove suitable for in vitro demonstrations of coronavirus infection of salivary glands. Salivary glands are known to serve as reservoirs for various infectious diseases and have been identified as targets of severe acute respiratory syndrome coronavirus. To this end, Tanaka et al. [141] had successfully generated functional salivary gland organoids from hiPSCs by modifying the method used to induce organoids of salivary glands originating from mouse embryonic stem cells (Fig. 9C). These hiPSC-derived salivary gland organoids exhibited specific cell lineages, morphological characteristics, and physiological functions characteristic of salivary glands. The researchers had confirmed that coronavirus can infect and replicate within these salivary gland organoids. The in vitro model developed in this study provides a promising approach to investigate the salivary gland's role as a viral reservoir. hiPSC-derived salivary gland organoids, in particular, are a valuable tool for this purpose.

#### Sweat and sebaceous glands

**Fig. 10.**
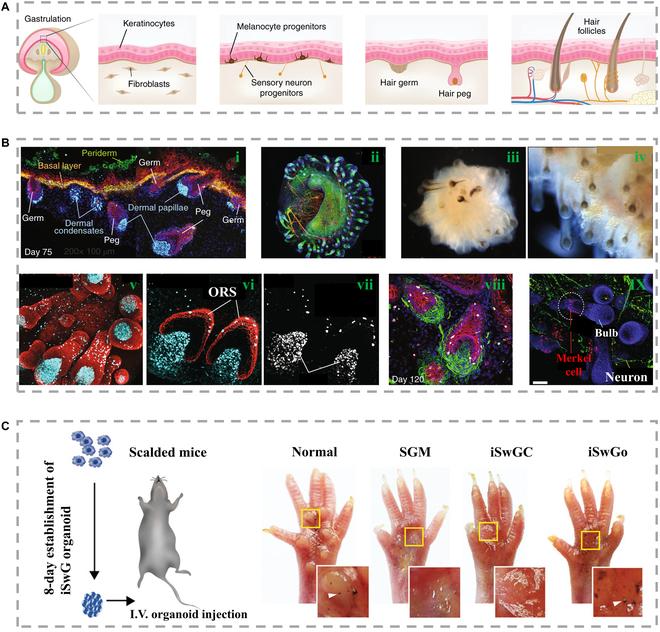
(A) The process of skin development in the body. The ectoderm produces the final germ layer, ectoderm, mesoderm, and endoderm [142], Copyright 2022, Springer Nature. (B) (i) Representative image of dermal condensates and dermal papillae. (ii) Representative immunohistochemistry images of skin organs on day 140. (iii) A depicted dark-field snapshot of skin organs derived from 125WA25hesc origin (left) and (iv) a perspective of human fetal facial skin tissue at 18 weeks (right). (v to vii) Noteworthy whole-mount immunostaining visuals of hair follicles encompassing dermal papilla and melanocytes on the 85th day. (viii) MITF also marked outer root sheath (ORS) fluid to counteract melanocytes onto epithelial cells. (ix) A comprehensive illustrative image capturing whole immunostaining of hair follicles within skin organs on the 110th day [142], Copyright 2022, Springer Nature. (C) Illustrated depiction outlining the procedural journey. The starch-iodine sweat test, conducted on the paw skin of mice subjected to heat injury, revealed a distinctive reaction solely in the paws of mice that had been treated with sweat gland organoids. This reaction became evident on the 21st day post-transplantation [143], Copyright 2021, Wiley-VCH.

The skin, being the largest and outermost organ of our body, plays a crucial role in protecting our body from external harms. This protective function is supported by its appendages, including hair follicles, sebaceous glands, sweat glands, and others (Fig. 10A) [142]. To date, the primary focus of in vitro skin-derived strategies has been on generating keratinocytes and fibroblasts from distinct cultures and then combining them to form a skin-like bilayer. Regrettably, these models fail to consider the inclusion of glands, which severely limits their biological and structural features. Consequently, designing a complete human skin model capable of generating all components, including appendages, continues to pose great challenges for the scientific community.

The sebaceous glands play a vital role in the skin's protective function by secreting oil into the ducts at the hair follicle junction, which aids in preventing water loss from the interfollicular epithelium and contributes to thermoregulation. To construct sebaceous glands, Lee et al. [142] have outlined a comprehensive procedure for cultivating hPSC populations into 3D in vitro culture systems, resulting in the development of hairy skin tissue (Fig. 10B). Through the implementation of precise culture conditions, PSCs were effectively directed to undergo differentiation into surface ectoderm. This ectoderm then gave rise to the epidermis and dermis within individual organoid units. With extended incubation, these skin organoids also proceeded to generate hair follicles. In the late stages of development, the skin organoids exhibited a highly biomimetic and complex structure, even including sensory nerves, and formed a cell composition and structure similar to fetal skin tissue at around 5 months. These skin organoids can be maintained in culture for up to 6 months. Additionally, they captured phase-contrast images of hair follicles in striking mouse skin organoids at different differentiation time points. Using these images, they were able to measure and quantify the length of the prominent pilosebaceous unit. This breakthrough makes organoids a useful tool for studying basic skin biology, model-ing diseases, and reconstructing or regenerating skin tissue in the future.

Patients with large skin defects often face challenges because of the limited number of stem cells present in the wound bed, which makes it difficult to form sufficient sweat glands. However, great progress has been made in patient-specific glandular tissue engineering by utilizing reprogrammed cells for regenerative therapy. In this regard, Sun et al. [143] presented a novel strategy for constructing human skin sweat glands in vitro (Fig. 10C). Initially, the researchers induced the expression of specific signals in human epidermal keratinocytes (HEKs) by binding them to a specific sweat gland medium, which efficiently converted the HEKs into sweat glands cells. These sweat glands cells exhibited the characteristic morphology, gene expression patterns, and functions similar to primary human sweat gland cells. Subsequently, sweat gland organoids with native sweat gland structure and biological properties were obtained by culturing sweat glands cells in a unique 3D culture system. Finally, these in vitro constructed sweat glands were successfully transplanted into a mouse skin injury model and developed into fully functional sweat glands in vivo. The regeneration of functional sweat gland organoids from reprogrammed HEKs represents a great advancement in personalized sweat gland regeneration and highlights the enormous translational potential of this approach for treating large skin defects.

#### Mammary

**Fig. 11.**
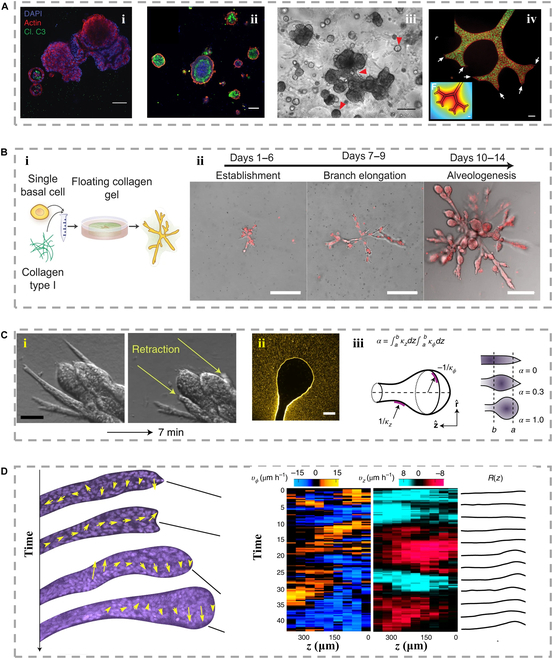
(A) (i and ii) Organoids stained with actin (red) and cysteamine pase3 (green) to show lumen clearance, or CK8 (green), CK14 (red), actin (white), and DAPI (blue) [144], Copyright 2021, Springer Nature. The culture image of (iii) multiple types of organ structure, Copyright 2021, Springer Nature, and (iv) breast branching morphology, Copyright 2004, Science. (B) (i) Illustration depicting the 3D culture framework; (ii) solitary primary human basal mammary epithelial cells were nurtured within a buoyant collagen gel environment in different times [146], Copyright 2021, Springer Nature. (C) (i) Alterations in displacement field due to cutting; (ii) organoids cultured within fluorescent collagen exhibit complete encirclement by a compacted collagen layer; (iii) for the purpose of delineating branch morphology, the introduction of the shape index (α) arises, characterized by axial and circumferential curvature [147], Copyright 2021, Springer Nature. (D) The observation of cellular dynamics during the formation of acini showed that the rotation was limited to the far end in space and was related to the position of the new alveoli [147], Copyright 2021, Springer Nature.

The morphological changes of mammary cells involve the occurrence of branching morphology, the formation of polar acini, etc. In vitro models have enabled the study of these phenomena, revealing the crucial role of different culture substrates in shaping the resulting morphologies (Fig. 11A, i and ii) [144]. For instance, mammary epithelial cells cultured in common matrigel tend to form acinar structures, whereas highly oriented collagen promotes elongation and branching morphogenesis of mammary ducts through the mechanical anisotropy generated by the fibrous domains. These observations were further supported by a micropatterning method developed by Nelson et al. in 2004. They determined that it is the geometry of the tubules that determines the location of the branches, while the sites with the lowest concentrations of local autocrine growth factors do not inhibit mammary morphology (Fig. 11A, iii and iv) [145]. To delve deeper into this phenomenon, Buchmann and colleagues employed models involving floating, attached, and partially detached collagen gels. These models were utilized to investigate the influence of the ECM on the morphogenesis of mammary epithelial cells (Fig. 11B) [146]. Their investigation unveiled a spatially and temporally controlled orchestration of epithelial morphogenesis within 3D collagen gels. Within this context, collagen emerged as a pivotal factor in the initiation of mammary duct formation and branching. Intriguingly, human mammary epithelial MCF10A cells embedded in collagen displayed acinar and tubular structures exclusively when the gel disengaged from the culture wells, facilitating cellular contraction. In stark contrast, MCF10A cells nurtured within attached gels adopted a sheet-like arrangement, reminiscent of epithelial cell structures grown on plastic surfaces, rather than those encountered within breast tissues. These findings emphasize the importance of carefully choosing culture substrates and geometry in in vitro models to accurately recapitulate mammary gland morphogenesis, providing valuable insights into the underlying mechanisms of this complex process.

The use of organoid technology has enabled the generation of ample quantities of breast cancer organoids, while human mammary epithelial cells can be cultured as organoid cultures without requiring immortalization. 3D in vitro cell culture models have been employed to deconstruct the structural and functional mimicry of mammary epithelial tissue. In a recent study by Rosenbluth et al. [144], it was observed that mammary epithelial cells exhibited self-organization into multiple structural types within organoid cultures. Plenty of the structures were observed to be acinar, possessing a lumen that was either isolated or associated with budding organoids. Additionally, solid spheres were also detected alongside branched tubular structures. The occurrence of branching or budding structures was noted in only 1 out of 102 organoids. The latter was seen predominantly in early passages, becoming less frequent with repeated dissociation and extended growth. Luminal cells, basal cells, or a combination of both cell types were found to be present within the organoids, indicating that the culture retained both basal and luminal cells.

Biomechanical factors, specifically stiffness, play a crucial role in limiting organoid generation. Epithelial cells struggle to form round and polar acinar structures in a 3D environment. Furthermore, differences in the spatial distribution of acini and ducts within the gel in matrices with differences in stiffness suggest that mechanical factors also influence the formation of mammary cell morphology (Fig. 11C and D) [147]. Collagen fibrous organization also contributes to epithelial morphogenesis, with cells most likely organizing collagen fibers by applying traction in early stages. This differentiated fibrous organization ultimately controls the formation of ductal or acinar structures by determining epithelial morphology. While the progression from tubular ducts to terminal spherical acini is a widespread occurrence in the developmental journey of advanced organisms, the mammary gland stands out as a unique illustration. It relies on distinct hormonal triggers across various developmental phases to orchestrate alterations in acinar configuration. This transformation, at its core, stems from a localized shift in intracellular tension within evolving human mammary organoids—shifting from anisotropic to isotropic tension. The morphological shift in alveolar generation within these organoids can be comprehended as a sprouting instability set in motion by a reduction in tension anisotropy. This intricate mechanism operates as a comprehensive feedback loop, ultimately influencing tissue shape through the lens of hydrodynamics.

## The Application of Biomimetic Gland Models

Biomimetic gland models have important potential for both basic and applied research. They replicate the intricate structural and functional characteristics of native glandular tissue, providing crucial insights into the cellular and molecular mechanisms governing gland development, function, and disease [148,149]. Specifically, they are useful for investigating the impacts of environmental factors, genetic mutations, drug treatments, toxins, pathogens, and other stressors on gland function [150]. Recent advancements in microfluidic systems and organ-on-a-chip technologies have introduced new bioinspired engineering approaches to miniaturize these interfaces. These advancements facilitate meticulous in vitro investigations into the orchestrated interplays among parenchymal and stromal cells within controlled surroundings. The progress in biomimetic engineering offers new prospects for studying glandular tissue and its complex interactions with the microenvironment in vitro, which can improve our comprehension of glandular development, function, and disease. Furthermore, these models are valuable for screening potential therapeutic agents for glandular diseases such as cancer, diabetes, and endocrine disorders, as well as for studying complex signaling pathways and crosstalk between different cell types within glandular tissue.

### Modeling diseases

**Fig. 12.**
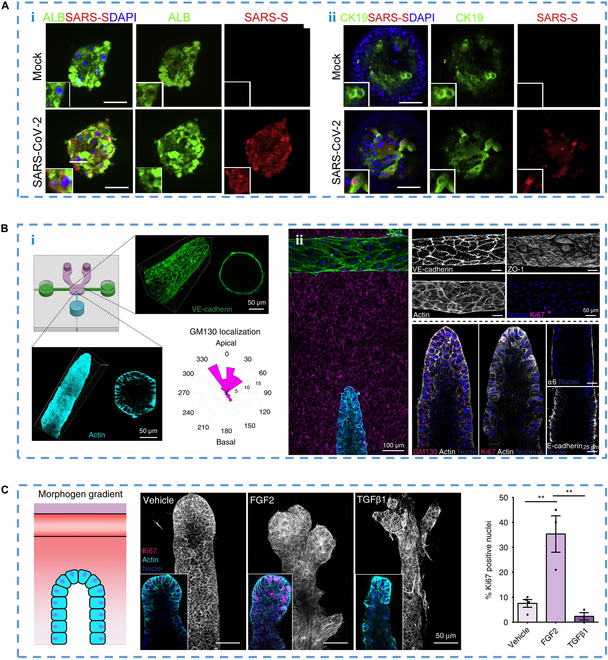
(A) (i) Confocal imaging of SARS-CoV-2 virus infected 24 h post-infection in adult hepatocyte-like organs. (ii) Confocal imaging of human bile duct cell-like organs when SARS-CoV-2 virus infects 24 h post-infection [152], Copyright 2021, Springer Nature. (B) (i) Schematic diagram of engineering mammary gland construction composed of 2 types of cells (endothelial cells and lumen cells). (ii) A micrograph presenting the maximum intensity projection of the combined suture situated at the base of the tissue within the mammary epithelial duct (displayed in cyan, representing adiponectin) and neighboring vessels (depicted in green, also representing adiponectin) [153], Copyright 2021, Springer Nature. (C) The structural gradient orchestrated by growth factors via non-cellular vascular conduits [153], Copyright 2021, Springer Nature.

Animal models' inability to accurately predict treatment responses in humans raises concerns about their use in basic research. During the COVID-19 pandemic, researchers developed in vitro physiological models to replicate the effects of the SARS virus on various organs and glands [151]. Yang et al. [152] utilized directed differentiation of hPSCs to generate pancreatic endocrine cells and other organoids representing all 3 germ layers (Fig. 12A). Infected hPSC-derived pancreatic endocrine cells exhibited upregulation of apoptosis-associated genes and downregulation of cell survival-associated genes in response to SARS infection. The results suggested that SARS-CoV-2 induced chemokine induction in pancreatic endocrine cells, similar to what is observed in COVID-19 postmortem samples. The researchers also successfully infected primary human organoids mainly composed of cholangiocytes with SARS, providing evidence of the model's efficacy in studying the effects of diseases on human organs. Thus, this study successfully established an in vitro biomimetic gland model for investigating the impact of diseases on human organs, mitigating the issue of relying solely on animal models in basic research**.**

The mammary gland, a notably dynamic and well-vascularized tissue undergoing subsequent transformations in both regular developmental processes and disease conditions, demands a thorough examination of the intricate interplay between epithelial cells and the vascular system at the molecular and cellular levels. Co-culturing glandular epithelial cells with other cell types, such as stromal or endothelial cells, can provide researchers with a more comprehensive understanding of the complex interplay among these cell types and their contribution to glandular function. Kutys et al. [153] developed a 3D microfluidic platform that allows for the co-culture of human mammary ducts and perfused endothelial vessels (Fig. 12B and C). The framework faithfully replicated the consistent structural attributes of natural mammary tissue and its capacity to engage in diverse manifestations of branching morphogenesis. Using this system enables the simulation of mutations in specific disease signaling pathways, facilitating the observation of dramatic differences in morphogenetic and behavioral outcomes. Unlike traditional 3D acinar models where cells aggregate to form empty acini that undergo apoptosis, the recently devised platform stands apart. It adeptly captures the evolution and invasive tendencies of mammary epithelial cells across different developmental stages. This innovation provides a visual avenue for tracking alterations in cellular and tissue arrangements, the dynamics of tissue branching and invasion, and the functional transformation of the vasculature. Impressively, the model accurately reproduces distinct morphogenetic phenotypes in response to specific soluble growth factors, mirroring real-world observations and other systems. While the partial apical maturation of MCF10A cells might contribute to these outcomes, the exclusive morphogenic responses tied to growth factor sensitivity hint at an inherent diversity within mammary gland morphogenesis, further enriching our grasp of mammary gland development and the mechanisms governing its control in various diseases.

### Drug screening

Advances in high-throughput screening for drug toxicity, drug response, and metabolic disease have been facilitated by the development of functional human stem organoids [154]. The in vitro gland model provides a valuable tool for evaluating the efficacy and toxicity of drugs, especially those that may affect glandular function [155–157]. Traditional cell culture systems lack the complex interactions and functional characteristics of native tissues, making them unsuitable for drug evaluation [158]. Engineered in vitro gland models, on the other hand, aim to recapitulate the architecture, cell composition, and functional properties of glandular tissues in a controlled laboratory setting. For instance, the lack of liver biopsies from patients with liver diseases, as well as suitable cell and animal models, hampers targeted drug strategies for corresponding diseases. As mentioned before, hepatic organoid systems have become attractive models for preclinical or clinical drug screening due to their similarity to liver tissue and their ability to screen drug effects on liver phenotypes before testing in patients [159–161].

**Fig. 13.**
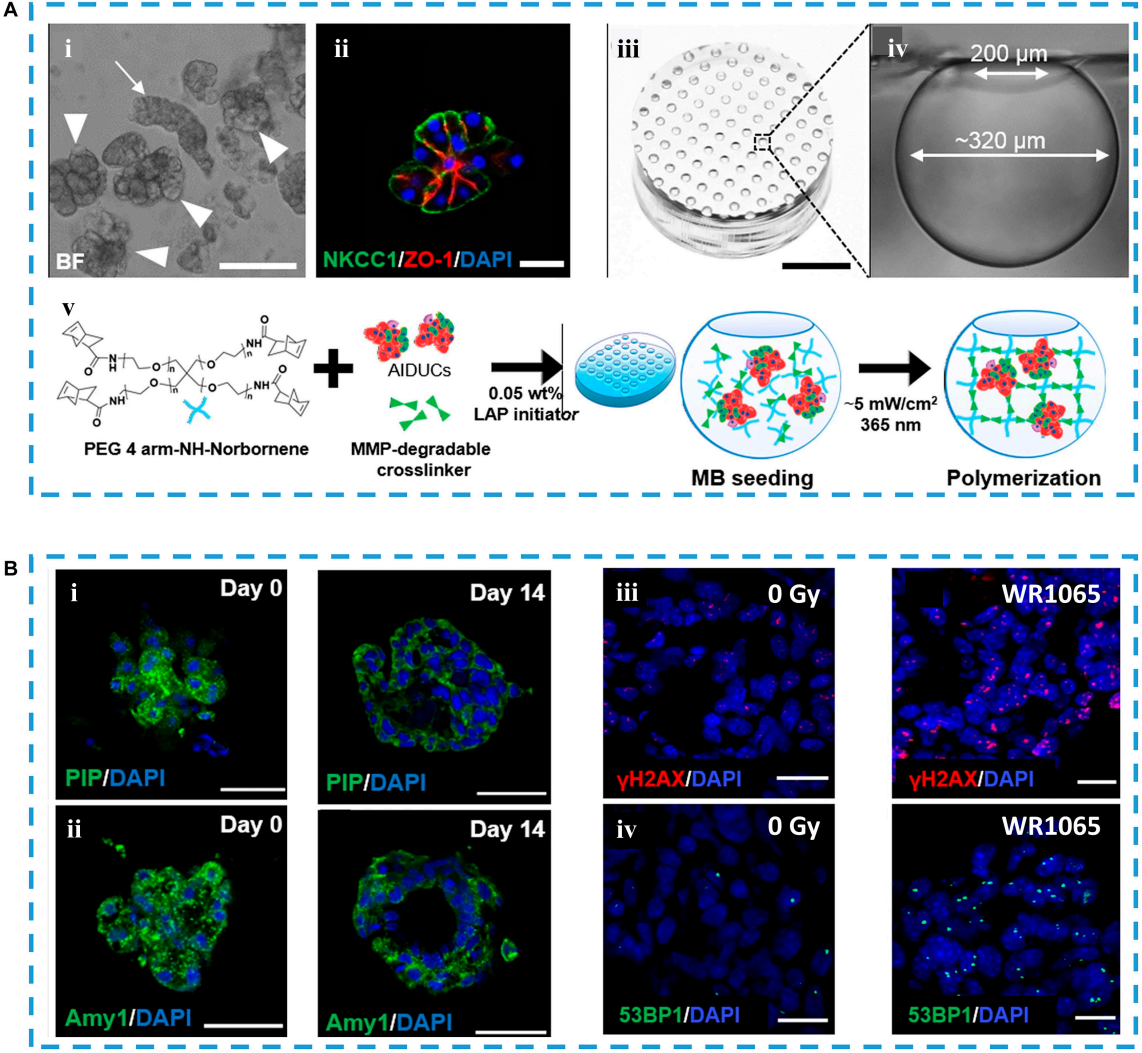
(A) Images of (i) isolated acinar cell clusters, (ii) immunofluorescence staining images with arrows indicating the striated duct complex, (iii) image of microbubble chip, and (iv) a cross-sectional depiction capturing the singular microbubble. (v) An illustrative diagram outlining the encapsulation of hydrogel within the chip configuration [163], Copyright 2021, Springer Nature. (B) (i) Prolactin-inducible protein immunohistochemical staining on day 0 and day 14, and (ii) immunohistochemical staining of amylase (Amy1) on day 0 and day 14; (iii) IHC staining showed the effect of irradiation and (iv) amifostine thiol on DNA damage (p53-binding protein) [163], Copyright 2021, Springer Nature.

Reduced salivation is a common side effect of various medications, which can be worsened by co-administration of multiple drugs. Additionally, radiation-based cancer therapies can lead to permanent loss of saliva, with a side effect of permanent impairment on salivary gland function. However, there is currently no appropriate in vitro model for conducting salivary gland experiments within normal radiation dose ranges. Additionally, formulating defensive strategies to avert the swift decline of the secretory acinar cell phenotype during cultivation has been a focus. To address this challenge, Song et al. [162] introduced a modular salivary gland tissue chip platform (Fig. 13A), which maintained the secretory acinar cell phenotype in vitro and provided a controllable microenvironment for long-term cell culture. These chips have been utilized for radiation response studies and for screening radioprotective drugs to alleviate xerostomia. Importantly, this methodology stands independent of stem cells or iPSCs, given that protocols for deriving salivary gland cells from iPSCs are still in the developmental phase. The outcome of this endeavor is a salivary gland tissue-on-a-chip system, which proves invaluable for conducting extensive and detailed screenings. Its application extends to mechanistic investigations and drug evaluation.

Parathyroid disease is a pathological condition characterized by abnormal calcium homeostasis and disturbances in parathyroid hormone secretion. The use of in vitro models may facilitate targeted therapy of parathyroid disease. In one study, Noltes et al. [163] created human parathyroid organoids to mimic human parathyroid tissue (Fig. 13B). Various organoids have been obtained by isolating and culturing glandular cells. The model demonstrates responses and correlated responses to changes in calcium concentrations and drug treatments. Under normal physiological conditions, parathyroid hormone secretion is negatively regulated by increased extracellular calcium levels. When exposed to high levels of calcium, human parathyroid organoids showed a significant reduction in parathyroid hormone secretion, compared to normal culture conditions. Subsequently, when human parathyroid organoids returned to basal calcium levels, a significant increase in parathyroid hormone secretion was observed. These findings suggest that human parathyroid organoids have a complete functional response. To assess the usefulness of patient-derived human parathyroid organoids in testing new therapeutic targets, validated therapeutic compounds were used to assess drug effects on human parathyroid organoid secretion. The results demonstrated that parathyroid hormone secretion was significantly reduced in parathyroid organoids with the treatment of various concentrations of cinacalcet compared to dimethyl sulfoxide controls. Additionally, the parathyroid organoids effectively recreated the genetic and protein profiles of the native tissue. This model provides a useful tool for investigating potential therapeutic targets and imaging agents for parathyroid diseases.

### Tissue regeneration

The in vitro construction of glands for tissue regeneration is a promising approach for addressing glandular dysfunction caused by injury, disease, or aging [164]. One approach to glandular tissue regeneration is tissue engineering techniques, which involve the combination of cells, scaffolds, and signals to create functional tissue substitutes [165]. Tissue engineering glands incorporate the isolation and expansion of glandular cells, followed by the seeding of these cells onto a scaffold that mimics the architecture of the native gland [166]. The scaffold can be made from natural or synthetic materials with a design to provide mechanical support [167], promote cell adhesion and proliferation, and allow for the exchange of nutrients and waste products [168]. Several studies have successfully employed tissue engineering to regenerate different types of glands, including salivary glands, lacrimal glands, and mammary glands [169]. For example, researchers have used 3D printing technology to create customized hydrogel scaffolds that promote the formation of functional acinar units in cultured human salivary gland cells. Other approaches have involved the use of decellularized glandular tissue as a scaffold, which provides a natural microenvironment for seeded cells to regenerate functional glandular tissue.

**Fig. 14.**
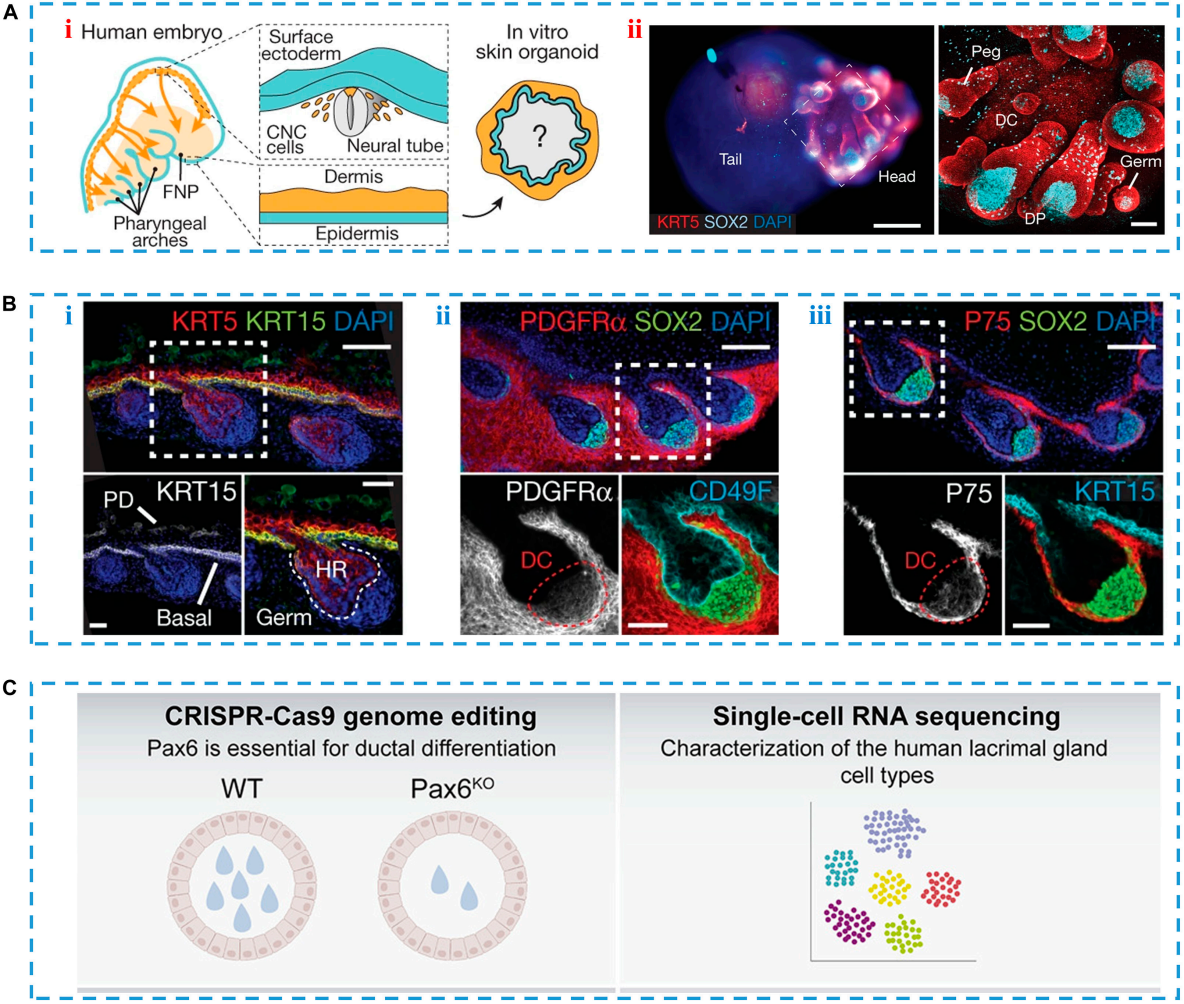
(A) Schematic diagram of (i) skin organoid construction and (ii) skin organ sample on day 85 [170], Copyright 2020, Springer Nature. (B) Immunostaining of skin organoid and hair substrate on the 75th day. IHC images of (i) epidermis and pericortex, (ii) dermis, and (iii) dermal condensate cells. Dashed box is the enlarged area [170], Copyright 2020, Springer Nature. (C) Organoid applications used as the research object of lacrimal gland in vitro [171], Copyright 2021, Cell Press.

In the field of tissue engineering, the reconstruction of appendage skin in both cultured cells and bioengineered transplants has shown promising results, particularly in the use of in vitro gland construction. The researchers developed a model to study how different glandular structures in human skin change during development (Fig. 14A) [170]. Among them, the hair follicle models can reproduce the same change process of hair follicles with that of human development. In addition, these cutaneous organs are periodically distributed throughout the epidermis, indicating the retention of accessory structure patterning mechanisms (Fig. 14B) [170]. However, after about 150 days of cultivation, accumulation of epithelial cells in the central part of the skin organ and abnormal morphology of hair follicles were observed, suggesting that this time point may represent the limit of skin organ cultivation. Implanting constructed skin organoids with hair into incisions on the backs of nude mice showed that 55% of the xenografts developed hair, although about 22% did not develop sweat glands. Nonetheless, skin organ models can be used to simulate a range of genetic skin diseases and cancers, providing a more complete tissue-engineered skin model for drug screening. In addition, by manipulating other cell lineages, these skin organoid models could be used to recreate skin tissue in burn or wound patients.

The majority of tear secretion, pivotal in preserving the stability of the ocular surface, is attributed to tear glands. Despite this, there remains limited insight into the diverse cell makeup and origins of tear fluid components. To enhance our comprehension of lacrimal gland disorders and unlock the prospects of regenerative therapies, it becomes imperative to cultivate fully developed human lacrimal gland epithelial cells. Addressing this need, Bannier-Helaouet and collaborators successfully initiated the development of lacrimal gland ductal organoids using both mouse and human tissue samples. Notably, these organoids demonstrated responsiveness to neurotransmitter stimulation and exhibited post-transplantation expansion (Fig. 14C) [171]. Tissue and organoid analysis through single-cell mRNA sequencing has brought to light the intricate cellular diversity within the lacrimal gland. This newly created organoid system stands poised as a potent screening tool for identifying agents that induce tear flow, given its propensity to elicit water secretion upon neurotransmitter exposure. However, it is important to note that the current organoid model predominantly mirrors the ductal segment of the tissue. To attain a more comprehensive grasp of acinar cell regeneration, further enhancements are requisite, particularly in the development of organoids derived from acinar cells. In vitro construction of glands for tissue regeneration shows promise as an approach for the treatment of glandular dysfunction. By combining tissue engineering techniques with advances in stem cell biology and biomaterials, researchers can create functional glandular substitutes that may one day be used to restore glandular function in patients with various glandular disorders.

### Bioreactor

In vitro glands can be utilized in bioreactor systems to produce large quantities of functional glandular tissue for research, drug discovery, and therapeutic applications. Bioreactors provide a controlled environment in which cells and tissues can be cultured under conditions that mimic the in vivo microenvironment. One application of in vitro glands in bioreactors is in the production of glandular secretions for use in drug development and testing. For example, salivary glands can be cultured in bioreactors and stimulated to produce saliva, which can then be collected and used to test the efficacy of drugs designed to stimulate or inhibit salivary gland function.

The mammary gland bioreactor uses the mammary glands of transgenic animals to express genetically engineered medicinal proteins. It is currently the most widely used animal bioreactor with the most in-depth research, the most foreign protein production, and the earliest approval for commercial production in the world. The salivary glands of animals have the potential to be highly efficient bioreactors, because they can synthesize and secrete a variety of bioactive proteins into saliva throughout the animal's life. Salivary glands can specifically express some important exogenous protein factors and enzymes, such as nerve growth factor, so these exogenous target proteins can be obtained from the saliva of transgenic animals by constructing engineered salivary gland models. Zeng et al. employed the pig salivary gland as a bioreactor, using transformed pigs from gene editing technology. These transgenic pigs specifically express and secrete functional growth factor proteins at high levels in their salivary glands. Furthermore, they developed surgical and non-surgical methods to efficiently collect porcine saliva, and successfully isolated purified functional growth factor proteins from the collected saliva and confirmed their biological activities. This new approach to animal medicine holds promise for improving human health and biomedical research.

**Fig. 15.**
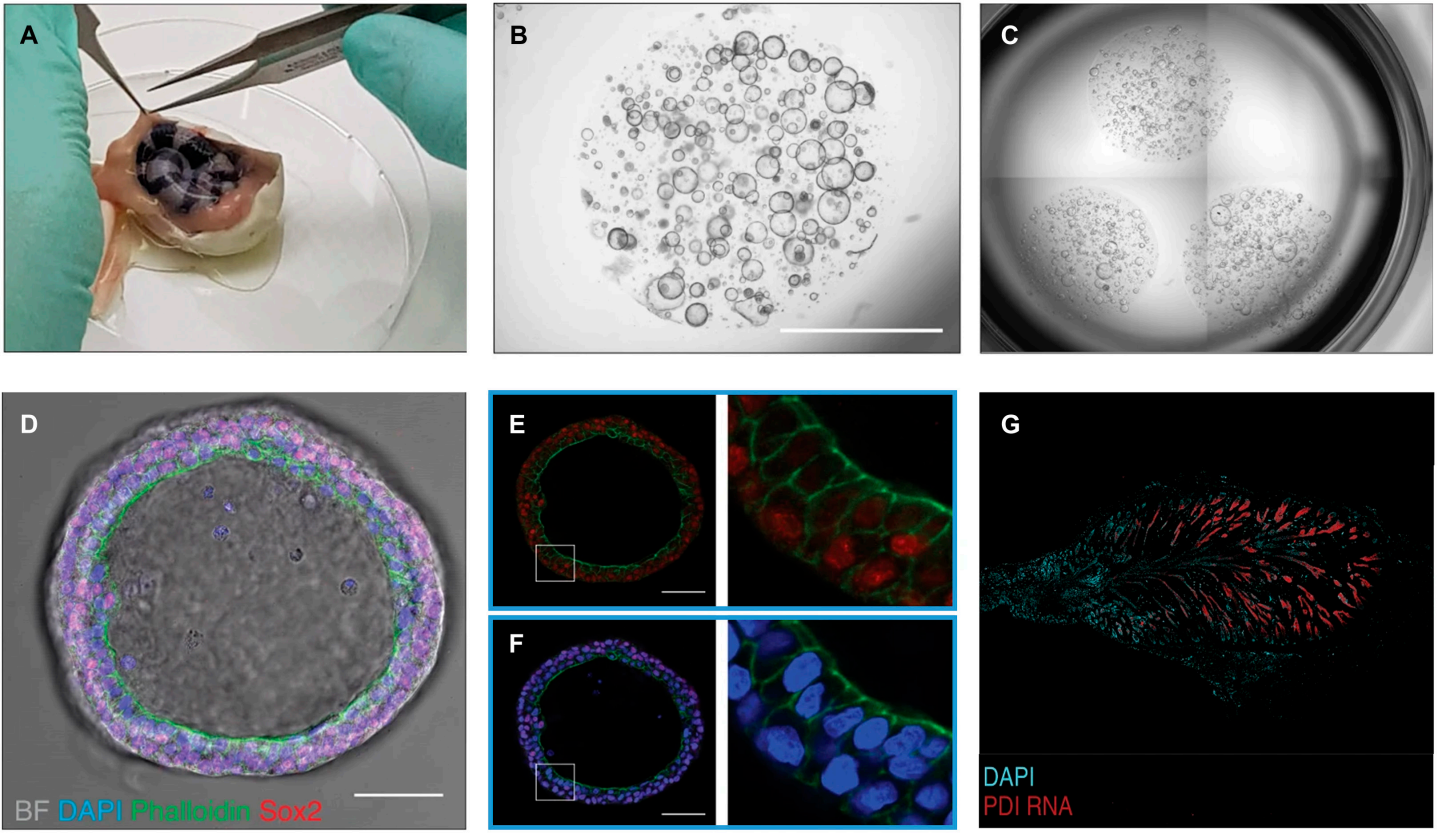
(A) Isolating the venom gland tissue from late-stage embryos (approximately 5 days prior to hatching) under sterile conditions. (B) Capturing a bright-field image of a diverse assortment of organs within the roughly 15-μl liquid droplet. (C) Presenting a well of a 24-hole plate alongside 3 liquid droplets of basement membrane extract from above. (D) Displaying a merged immunofluorescence image. (E) Depicting an immunofluorescence image showcasing SOX2 (in red). (F) Offering a confocal microscope image of F-actin (green, representative of stem protein) within the late embryo venom gland, followed by fluorescence in situ hybridization. (G) The color blue corresponds to DAPI, while the color red corresponds to PDI (peptide disulfide isomerase), a vital protein for toxin folding, RNA [172], Copyright 2022, Springer Nature.

The snake venom gland, a specialized epithelial organ dedicated to toxin production, holds dual significance: not only does it fulfill its toxin-producing role, but it also serves as a valuable reservoir for identifying bioactive compounds with potential in drug development. Each year, millions of people endure snakebite-related complications. Nonetheless, snake venom also stands as a prolific source of bioactive molecules, acknowledged or potentially promising for therapeutic use. Organ-like structures that closely resemble the snake venom gland can be created from embryonic specimens or freshly obtained glands of adult snakes via biopsies. Biopsies collected from late-stage embryos, following removal from the egg, offer a reduced risk of bacterial contamination during culture, as opposed to organoids originating from early-stage adult snake biopsies in the developmental stages of new organ-like structures. The protocol has been successfully applied using both late-stage embryonic and recently deceased adult snake glands. The resulting organoids preserve the cellular diversity inherent to the venom gland and can be manipulated concerning cell-type composition by modifying the culture medium. Puschhof et al. [172] offer a comprehensive guide on how to derive and nurture these organoids, how to dissociate them into individual cells, and how to freeze-preserve and steer their differentiation into entities capable of toxin production (Fig. 15). Furthermore, comprehensive instructions are included for carrying out essential downstream analyses, encompassing quantitative real-time PCR, bulk and single-cell RNA sequencing, and immunofluorescence and immunohistochemistry techniques.

### Personalized medicine

Cancer is a complex disease with considerable variation in staging, genetic background, and molecular behavior [173]. As an effective tool in capturing this heterogeneity, patient-derived organoids (PDOs) represent a more comprehensive model of human disease than a single genetic summary, providing a potential superior approach to clinical therapeutic decision-making compared to genomic guidance [174,175]. Patient-derived xenografts (PDXs) have been the specific model in cancer drug discovery and personalized medicine for decades [176]. They offer many benefits, including preservation of clonal architecture within tumors over repeated passaging, faithful recapitulation of patient drug response, and capture of genetic diversity of patient tumor types [177]. However, PDXs are both time-consuming and expensive, which greatly limit their further applications [178]. By contrast, PDOs offer superior cost-effectiveness, higher establishment success rates, and greater throughput. As larger-scale studies continue to establish the potential of PDOs in predicting drug responses and capturing patient heterogeneity, organoids are rapidly becoming one of the most powerful tools in our arsenal for simulating human physiology.

**Fig. 16.**
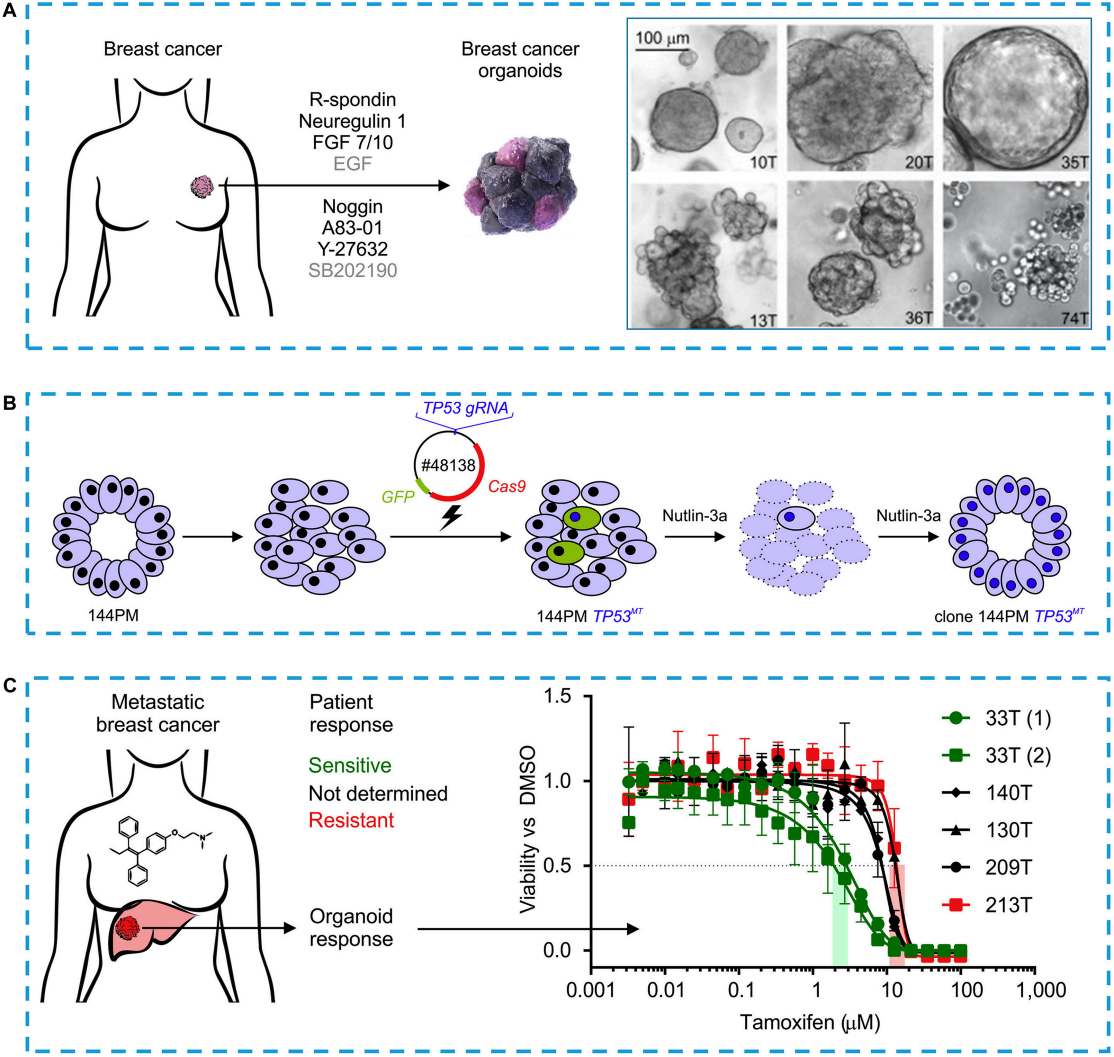
(A) Producing mammary organoid lines from primary mammary gland cells by employing a set of finely tuned medium constituents [181], Copyright 2018, Cell Press. Right: bright-field images depicting the phenotypes of major mammary organoids. On the top row, we observe dense and solid viscous organoids on the left and in the middle, while the rightmost image displays saccular and hollow organoids. On the bottom row, a progression is evident, with organoids becoming progressively more scattered from left to right [181], Copyright 2018, Cell Press. (B) Depicting the sequential stages in generating TP53 mutant mammary organoids, including dissociation, electroporation, selection, and the subsequent growth of clonal populations. (C) The schematic diagram shows the experimental setup of organogenesis from metastatic breast patients. The response of patients to standard care (tamoxifen here) was recorded and compared with the in vitro response (dose–response curve on the right side) of mammary organs derived, respectively [181], Copyright 2018, Cell Press.

In recent years, preclinical breast cancer research has heavily relied on cell lines to represent a complex and diverse disease affecting millions of patients [179,180]. Despite being useful for high-throughput screening, breast cancer cell lines have limitations in capturing the full spectrum of breast cancer and are not clinically relevant for individual patients. Addressing this challenge, Sachs and colleagues have devised a remedy by formulating culture parameters for human breast epithelial organoids. Their efforts yielded a remarkable outcome: a collection of more than 100 primary and metastatic breast cancer organoid cell lines, effectively encapsulating the expansive spectrum of the disease's diversity (Fig. 16A to C) [181]. Importantly, the organoids exhibit no significant deviation from the original tumors in any aspect, even after prolonged passage. Using classical Cas9 gene editing, functional TP53 mutant clones were generated in the mammary organoids, which were largely retained. The breast cancer organoids managed to infiltrate all important gene expression-oriented classification frameworks. Moreover, they facilitated drug screening within an in vitro setting and mirrored patient responses that aligned seamlessly with outcomes observed in vivo, as corroborated by xenograft experiments. To demonstrate the predictive potential of breast cancer organoids, 2 organoid cell lines were selected and their differential responses to drugs that block signaling were examined. The results showed that breast cancer organoids responded to model drugs similar to in vivo models, successfully demonstrating the feasibility of using organoids to mimic glandular diseases.

### Other applications

In vitro gland models can be used to study the biology of cancer and to develop new cancer therapies. For example, researchers can use in vitro models of prostate glands to study how prostate cancer cells interact with the surrounding tissue [182–184]. Interestingly, natural foods have shown potential in inhibiting cancer, and glandular models can be used to quickly screen their effects. For example, broccoli is a widely recognized vegetable for its nutritional value and chemoprotective potential [185]. In vitro models have been used to evaluate the effects of freeze-dried broccoli sprout extracts on normal and thyroid tumor cells, as well as their anti-inflammatory and antioxidant potential. To test the potential toxicity of broccoli sprout extract on normal nonneoplastic thyroid follicular epithelial cells, a preliminary in vitro assessment was conducted [185]. Results showed that even after prolonged exposure for up to 72 h, broccoli sprout extract was not toxic to non-neoplastic thyroid cells. This demonstrated the potential of broccoli sprouts to reduce thyroid cancer cell viability and prevented associated inflammation using an in vitro thyroid model.

Biomimetic bioartificial liver systems are emerging as a potential solution for end-stage liver disease, providing an alternative to liver transplantation [186,187]. This technology is similar to dialysis equipment for kidney disease patients, allowing for the exchange and renewal of metabolic waste back into the human body [188]. While still incomplete, this type of artificial liver system can offer patients with a bridge to transplantation for liver failure. Our team has developed a novel biomimetic bioartificial liver system by integrating microparticles and hollow fiber tubes loaded with stem-cell-derived hepatocytes into a miniaturized artificial liver platform [189]. This system mimics the functional unit of the human liver, the hepatic lobule, with microcarriers simulating the structure and semipermeable microtubules simulating large blood vessels. Compared to planar culture, experiments show that the cell retention rate on microcarriers is much higher and metabolic activity is enhanced. Further miniaturization of the equipment scale is expected to lead to better results, ultimately achieving a complete simulation of the human liver.

## Conclusions and Future Perspectives

In conclusion, biomimetic glandular models especially the glandular organoids have emerged as a powerful tool for recapitulating both the structural and functional aspects of glands in vitro. During the development process, stem cell research enables the utilization of ideal cell sources like patient-specific stem cells, while the microfluidic technique could provide a more realistic microenvironment for cell culture. Here, we have reviewed the progress of engineering strategies for accurate construction of gland models with similar structure, function, and microenvironment features to that of actual glands, from traditional 2D cell culture to novel organoid-on-a-chip technology. Based on different glandular models, their applications such as drug screening and disease modeling have been presented.

The development of organoid chip platforms for modeling glandular health and pathological phenotypes has been remarkable. However, their further advancement has been hindered by material complexity and safety concerns in their fabrication. The traditional photolithographic silicon technology suffers from the variability and inconsistency between different manufacturing lots and on-wafer capping models produced in different laboratories, while the commonly used polydimethylsiloxane (PDMS) poses a challenge in simulating the ECM environment of glands accurately. Therefore, it is essential to achieve a balance between safety and the need to create scalable, cost-effective commercial platforms, which may be realized by designing materials that can sufficiently mimic the complexity of natural substrates for controlling cell fate. Furthermore, to enable clinical use, it is crucial to identify materials that are free of any components derived from animals (Matrigel) and that can be fully characterized.

One of the primary concerns surrounding the development of gland models pertains to the creation of increasingly intricate glandular structures. While current models predominantly concentrate on relatively simple glandular tissues, such as the mammary gland or salivary gland, researchers are currently striving to fabricate more complex glandular tissue models, such as those of the pancreas, thyroid, and pituitary. This aspiration necessitates an accurate comprehension of the gland's biological growth and development processes from a biological perspective. Furthermore, a critical developmental domain is the adept integration of microfluidic systems into gland models, given their capacity to create more realistic tissue environments. The utilization of microfluidic systems enables researchers to analyze the effects of physical forces and chemical gradients on glandular tissues.

The utilization of 3D printing methods is expected to further advance the field of in vitro gland model development. A promising prospect for the application of 3D printing gland model development lies in its ability to generate models that faithfully replicate the structure and functionality of human glands. Additionally, 3D printing can facilitate the fabrication of gland models that are more intricate and elaborate than those generated by conventional methods. For instance, models that incorporate diverse cellular types and structures, or accurately mimic the glandular microenvironment, can be generated by 3D printing. It is anticipated that 3D printing technology will be increasingly leveraged in the future to create gland models that are more precise and tailored to individual patients, to be utilized in research and clinical field.
